# Pupil dilation as a marker of attention/effort in aging and mild cognitive impairment

**DOI:** 10.1002/alz.71180

**Published:** 2026-03-13

**Authors:** Alina Zhunussova, Clare Loane, Elif Kurt, Grazia Daniela Femminella, Sabrina Lenzoni, Millie Duckett, Martina F Callaghan, Nikolaus Weiskopf, Raymond J Dolan, Robert Howard, Emrah Düzel, Dorothea Hämmerer

**Affiliations:** ^1^ Department of Psychology University of Innsbruck Innsbruck Austria; ^2^ Institute of Cognitive Neuroscience University College London London UK; ^3^ Department of Neuroscience Aziz Sancar Institute of Experimental Medicine Istanbul University, Topkapı Istanbul Turkiye; ^4^ Department of Translational Medical Sciences University of Naples Federico II Napoli Italy; ^5^ Department of Imaging Neuroscience University College London Queen Square Institute of Neurology London UK; ^6^ Wellcome Centre for Human Neuroimaging Queen Square Institute of Neurology University College London London UK; ^7^ Department of Neurophysics Max Planck Institute for Human Cognitive and Brain Sciences Leipzig Germany; ^8^ Felix Bloch Institute for Solid State Physics Faculty of Physics and Earth Sciences Leipzig University Leipzig Germany; ^9^ Max Planck University College London Centre for Computational Psychiatry and Ageing Research University College London London UK; ^10^ Division of Psychiatry University College London, Maple House London UK; ^11^ Institute of Cognitive Neurology and Dementia Research Otto‐von‐Guericke University Magdeburg Magdeburg Germany; ^12^ German Center for Neurodegenerative Diseases University Hospital Magdeburg Magdeburg Germany; ^13^ Center for Behavioral Brain Sciences Otto‐von‐Guericke‐Universität Magdeburg Magdeburg Germany

**Keywords:** attentional modulation, cognitive effort, locus coeruleus, mild cognitive impairment, pupil dilation

## Abstract

**INTRODUCTION:**

Pupil dilation (PD) can be easily measured and reflects responses to subjectively salient or cognitively demanding events. It therefore holds promise as a cognitive marker, especially for individuals with mild cognitive impairment (MCI) or other neurodegenerative conditions with restricted abilities to respond in cognitive assessments.

**METHODS:**

We assessed PD during two tasks, an oddball task for investigating attentional allocation and a Simon task, which additionally allows for investigating cognitive effort in younger adults (YAs), older adults (OAs), and patients with MCI.

**RESULTS:**

PD is a useful marker for investigating attention and cognitive effort in MCI, as suggested by elevated PD to salient stimuli in particular of individuals with better attentional control in MCI patients, as well as YAs and OAs.

**DISCUSSION:**

Measurement of PD may serve as an easy‐to‐administer measure to assess changes in cognitive function in healthy aging and MCI.

## Highlights

- PDs were recorded in patients with MCI during an oddball and Simon task.
- Elevated PDs during higher executive control suggest PD as a useful cognitive measure in MCI.
- Interindividual differences in PD in MCI explain differences in response speed in executive tasks.

## 1 BACKGROUND

Changes in pupil diameter reflect changes in cognitive functions such as attention, effort, or arousal.^1^, ^2^ Pupil size increases in response to salient stimuli that capture attention^3^, ^4^, ^5^ or task‐relevant stimuli that require a stronger a priori or top‐down attentional focus.^6^, ^7^ A common paradigm for inducing attentional pupil effects is the oddball task, where pupil size changes as participants respond to targets while ignoring irrelevant stimuli.^8^ Pupil dilation (PD) can also index cognitive load or (motor) effort,^2^, ^6^ increasing with more effortful conditions in a working memory task,^9^, ^10^ or as an indicator of effort and internal state changes during motor tasks.^11^, ^12^

While the full picture of the underlying mechanisms of cognition‐related PD in humans is still unclear, recent studies point to subcortical neuromodulatory systems as a potential contributor. The rapid phasic PD during cognitive tasks aligns with phasic activations in the noradrenergic locus coeruleus (LC).^13^, ^14^ Stimulating the LC in monkeys has been shown to elicit a phasic PD,^13^, ^15^, ^16^ strongly linking LC firing to PD. LC imaging studies in humans^8^, ^17^, ^18^ and animals^19^, ^20^, ^21^ further suggest its involvement in attentional and effort‐related events. However, stimulation of other brain areas also results in phasic PD, suggesting the link between LC firing and PD is not exclusive.^13^ Functional magnetic resonance imaging (MRI) studies show PD correlates in multiple brainstem centers,^22^, ^23^ leaving the specific PD‐controlling (brainstem) structures uncertain without targeted animal studies. Moreover, mechanisms identified in animals may not fully translate to humans. Given the small LC size and brainstem location, only a few human neuroimaging studies have examined the relationship between LC activity and pupil to test animal‐derived theories.^8^, ^22^, ^23^, ^24^ Murphy^8^ found increased LC activity for rare versus standard stimuli in an oddball task, supporting its role in attention‐related PD in humans. Future pharmacological imaging studies may clarify brain structures essential for controlling PD in humans.

The neural substrates of PD in animals and humans are not fully understood. Nonetheless, PD consistently reflects distinct cognitive processes such as salience detection, attention allocation, and effortful processing. Pupillometry offers a low‐burden, informative method for cognitive assessments in older adults (OAs) with mild cognitive impairment (MCI) or Alzheimer's disease (AD), usable at rest and during tasks. PD complements behavioral measures by indexing required attention or effort and revealing whether individuals differ in effort amount or show non‐selective executive recruitment.^25^ Millisecond‐resolution PD recordings can differentiate early versus late responses to cognitive events and distinguish multiple processes affecting a PD response (e.g., emotional salience and memory recognition).^26^, ^27^ Understanding processes underlying MCI performance therefore enables subtype differentiation, disease progression prediction, and intervention monitoring.

Despite its potential for populations with reduced cognitive capacities, few studies have used PD to characterize MCI populations, mainly examining cognitive decline in working memory and visual search and yielding mixed results. Some suggest PD responses to cognitive load differentiate MCI subtypes: Amnestic MCI showed greater PD at low to moderate loads than controls and non‐amnestic MCI. Under high load, PD declined in all groups, with a steeper decrease in non‐amnestic MCI than amnestic MCI or controls. Multidomain MCI failed to modulate dilation across loads.^28^ One study with sequence memorization reported reduced PD in AD patients versus controls, but not in MCI.^29^ In visual search, PD remained unchanged in AD compared to controls,^30^ whereas in another study, pupil response velocity decreased.^31^ Conversely, AD patients showed increased PD relative to controls in a non‐verbal auditory task.^32^ These differences may stem from varying disease stages (MCI and AD) and task demands. Additionally, better‐perfroming MCI patients showed higher task‐related PD, suggesting greater cognitive resource recruitment.^33^ Although attentional/executive deficits are early MCI/AD signs^34^, ^35^, ^36^ predicting AD progression,^37^, ^38^, ^39^ PD investigation of executive attentional control in MCI remains unexplored. We aimed to assess whether MCI patients effectively applied attention to relevant stimuli compared to healthy elderly and whether differences in allocated attention/effort inform task performance in MCI. Pertinent prior findings are mixed: AD patients showed exaggerated pupil responses to targets versus distractors,^40^ suggesting increased attentional control, while MCI patients showed reduced cognitive control in an antisaccade task.^33^

## 2 METHODS

### 2.1 Aims and hypotheses

This study makes several novel contributions to the field. First, we explore whether PD indicates the same pattern of allocated attention in MCI as in age‐matched controls and younger adults (YAs), that is, larger responses to relevant oddball stimuli,^8^ across two stimulus modalities (visual and auditory). Second, we examine PD in a Simon task, which allows for investigating the cognitive effort required to exert inhibitory control when processing incongruent response inputs.^41^ Our multitask approach, therefore, allows us to determine whether the sensitivity of cognitive processes reflected in PD varies across distinct executive processes in MCI. Finally, we integrate structural LC imaging using neuromelanin‐sensitive MRI with task‐based PD to understand whether interindividual differences in LC degeneration are associated with PD or cognitive function in aging and MCI, as suggested by recent studies.^42^, ^43^, ^44^ While our main focus is on comparing PD in age‐matched healthy OAs and MCI patients, we have also included a somewhat smaller sample of healthy YAs to serve as a reference for age‐related differences in PD.

**RESEARCH IN CONTEXT**

1. **Systematic review**: Using sources such as PubMed, the authors conducted a review of existing studies suggesting that PD can be used to assess allocation of attentional resources and cognitive effort and may be a tool for cognitive characterization in Alzheimer's patients. The current study used two executive processing tasks to answer whether pupillometry can serve as a valid tool for assessing top‐down attentional modulation or cognitive effort in patients with MCI.
2. **Interpretation**: Pupillary responses can be successfully recorded in MCI patients during the oddball and Simon tasks. Our results support the notion that PD measurement is an effective non‐intrusive measure for cognitive assessments in MCI.
3. **Future directions**: Future research should attempt to use pathological markers as diagnostic criteria to yield a more stratified MCI sample to validate the observed PD patterns of attentional modulation and cognitive effort with regard to disease severity in MCI.

We make the following predictions:

1. OAs and patients with MCI show a similar pattern of pupillometric effects across task conditions, suggesting a similar degree of engagement of PD in cognitive processing during two attentional and cognitive control tasks.
2. For the oddball task assessing attentional allocation, we expect an increase in PD for the oddball compared to the standard stimuli^45^ across both (auditory and visual) modalities, reflecting top‐down attentional modulation to infrequent stimuli. Given the known decline in attentional executive control in MCI,^46^ we expect that this effect will be greater in OAs compared to MCI. Similarly, we hypothesize that OAs will demonstrate better behavioral performance than MCI patients, with higher accuracy rates and faster reaction times (RTs).
3. For the Simon task assessing cognitive control, we expect increased pupil responses to incongruent as compared to congruent stimuli reflecting greater top‐down cognitive effort required to resolve stimulus‐response conflicts. Given the age‐related decline in executive functions,^47^, ^48^ we hypothesize that the ability to engage attention and mobilize cognitive resources as evident in PD should be comparatively reduced in MCI compared to OAs. Moreover, both OAs and patients with MCI are expected to show the classic Simon effect, with longer RTs and lower accuracy for incongruent compared to congruent stimuli, with this effect being more pronounced in MCI patients compared to OAs.
4. Within groups and tasks, we expect that greater PD, reflecting greater attentional focus or cognitive control, will be positively related to behavioral performance, that is, shorter RTs and higher accuracy rates across individuals. In addition, higher LC integrity is expected to correlate with better cognitive performance and greater PD in OAs and MCI patients.

### 2.2 Participants

The data presented here is part of a larger study including behavioral, PD, functional MRI (fMRI), and structural MRI assessments. A total of 93 participants took part in the larger study, including 30 YAs, 30 healthy OAs, and 33 patients with MCI (see Table S1 in the Supplement). As the main focus of the study was to compare OAs and age‐matched MCI participants, YAs were not routinely included in the PD recordings, especially for the oddball task. Therefore, whenever the number of participants in YAs was too small for statistical assessment, we report the results only descriptively to allow for an assessment of the typicality of the effects in comparison to existing pupillometry results in YAs. YAs were recruited via email from a participant panel at the University College London Institute of Cognitive Neuroscience. OAs were recruited via advertising and clinical teams when accompanying relatives or friends with MCI/AD to a memory clinic. MCI patients were recruited through the Join Dementia Research participant database and local memory clinics at Camden and Islington Healthcare Trust and North East London Healthcare Trust. All participants were right‐handed, had normal or corrected‐to‐normal vision, and had no history of psychiatric disorders. MCI in patients was confirmed through neuropsychological evaluation, that is, a score at least 22 or above on the Mini‐Mental State Examination (MMSE)^49^ as well as a diagnosis by a trained clinician. Healthy OAs and MCI patients were age matched and had to be within the age range from 60 to 85 years. YAs were between 20 to 30 years old. Caregiver‐rated assessments were obtained for 27 of the 33 MCI patients, as six were not accompanied by caregivers. The mean age of caregivers was 67.81 years (SD = 0.42; age range = 35 to 84), with six males and 21 females.

As detailed below, data from several participants had to be excluded from the analysis for four main reasons: (1) technical issues with the eye tracker, resulting in missing or unusable data; (2) difficulties in completing tasks, especially in MCI patients; (3) insufficient PD data due to excessive artifacts (e.g., blinking) in the case of the oddball task; and (4) challenges of PD acquisition in the magnetic resonance environment in the case of the Simon task related to insufficient signal strengths in infrared recordings.

Specifically, for the auditory and visual oddball tasks, we had to exclude 16 OAs (12 due to technical issues with the eye tracker set‐up in the eye‐tracking lab (hardware malfunctions, specifically issues with connection cables in the eye‐tracking lab), resulting in missing or unusable data, two due to difficulty in completing the task, resulting in no behavioral data being recorded, and two due an insufficient number of PD trials (less than three in one condition) due to excessive artifacts (e.g., blinking) in the PD data in one or both of the tasks); 10 patients with MCI (five due to technical issues with the eye tracker, four due to an insufficient number of PD trials, and one due to difficulty in completing the task; five YAs were excluded from statistical analyses, two because of insufficient numbers of PD trials, two due to technical issues with the eye tracker, and one due to difficulty in completing the task. Given the limited recruitment of YAs, this resulted in a small subsample (*n* = 7) of YAs for the oddball tasks and their data being presented descriptively for reference only. The final sample for both the visual and auditory oddball tasks included 14 healthy OAs and 23 individuals with MCI.

PD during the Simon task was acquired concurrently to fMRI assessments (not reported here). PD assessments were therefore acquired in the whole sample of 93 participants. However, PD is more challenging to acquire in the MRI due to several factors. Eye‐tracking quality is often compromised due to (1) shadows from the head coil that obscure pupil recordings depending on the position of the head in the coil and (2) a weaker pupil signal due to the position of the illuminator and camera behind the scanner bore compared to standard laboratory eye‐tracking set‐ups. In addition, head movements during recording can interfere with pupil signal acquisitions. For the Simon task, across the whole sample of 93 participants, a total of 31 participants were excluded: eight YAs (six due to challenges with acquiring PD in MRI setting, resulting in missing or unusable data, one due to difficulty in completing the task, and one due to unusually slow performance [RTs greater than three standard deviations from the group mean]), 10 OAs (eight due to challenges with acquiring PD in the MRI setting and two due to difficulty in completing the task), and 13 patients with MCI (10 due to challenges in acquiring PD in the MRI setting and three due to difficulty in completing the task). The final sample for the Simon task consisted of 22 healthy YAs, 20 healthy OAs, and 20 individuals with MCI.

Although sample sizes are at the lower bound, low sample sizes are not uncommon in eye‐tracking studies,^29^, ^30^, ^32^ which exhibit typically reliable PD effects in well‐established attentional and cognitive control tasks as employed here.^8^, ^50^ Moreover, to explore whether our study might have yielded spurious results given small and heterogeneous sample sizes, we explore the consistency of PD effects across individuals per group (Figures S1–S3 for the oddball task and Figures S4 and S5 for the Simon task in the Supplement). On the oddball tasks, larger PDs for oddball as compared to standard stimuli were observed in 92% to 100% of YAs, OAs, or those with MCI patients; on the Simon task, PD was larger for the incongruent condition in 68% of YAs, 85% of OAs, and 90% of MCI patients. The high consistency of these well‐established PD effects therefore suggests that our results are representative despite somewhat reduced sample sizes.

Finally, age matching was reconfirmed after exclusions for both the oddball task, *t*(29.89) = −1.61, *p* = 0.11, with mean ages of 69.92 and 73.91 for OAs and MCI patients, respectively, and the Simon task, *t*(37.63) = −0.84, *p* = 0.40, with mean ages of 71.2 and 73.2 years for OAs and MCI patients, respectively.

### 2.3 Procedure

Participants and/or caregivers completed neuropsychological and clinical questionnaires in a first session prior to MRI and PD assessments. All participants completed the National Adult Reading Test,^51^ Addenbrooke's Cognitive Examination (ACE),^52^ including the MMSE. For clinical assessments, YAs and OAs completed the Hospital Anxiety and Depression Scale^53^ and the Pittsburgh Sleep Quality Index (PSQI).^54^ MCI patients and/or caregivers completed the PSQI, Bristol Activities of Daily Living,^55^ Cornell Scale for Depression in Dementia,^56^ and the Neuropsychiatric Inventory.^57^ The ACE is a cognitive test designed to assess cognitive function in five domains, with higher scores indicating better cognitive function: attention (18), memory (26), verbal fluency (14), language (26), and visuospatial ability (16). A total score of 85 or less indicates cognitive impairment. The revised version of the ACE (ACE‐R)^58^ includes the MMSE, with a total score of 30. Scores below 24 may indicate cognitive impairment. In this paper, we present results for two domains of the ACE‐R – attention/orientation and memory – as well as the MMSE because of their diagnostic relevance to MCI, since these tests target cognitive domains known to be affected in the pathological progression of AD.

PD during the Simon task were recorded in the MRI scans, together with an emotional memory task (not reported here). The oddball tasks were performed on the afternoon outside of the MRI in an acoustically shielded room with controlled illumination. Each participant was reimbursed with £10 per hour for the time spent. Written informed consent was obtained from all participants and their carers prior to participation in the experiment. The study was conducted in accordance with the Declaration of Helsinki, and ethical approval was granted by the local Ethics Committee (University College London ethics reference number 17/0091).

### 2.4 Eye‐tracking recording

Pupillometric data were recorded using a laboratory‐based (oddball task) or scanner‐mounted (Simon task) infrared eye tracker (EyeLink 1000, SR Research). Participants' right eye pupil size was tracked at 1000 Hz and illumination levels of 100%. The lighting in the test room remained constant throughout the tasks. The eye tracker was calibrated prior to each task using a 6‐point or 9‐point calibration.

### 2.5 Tasks

**FIGURE 1:**
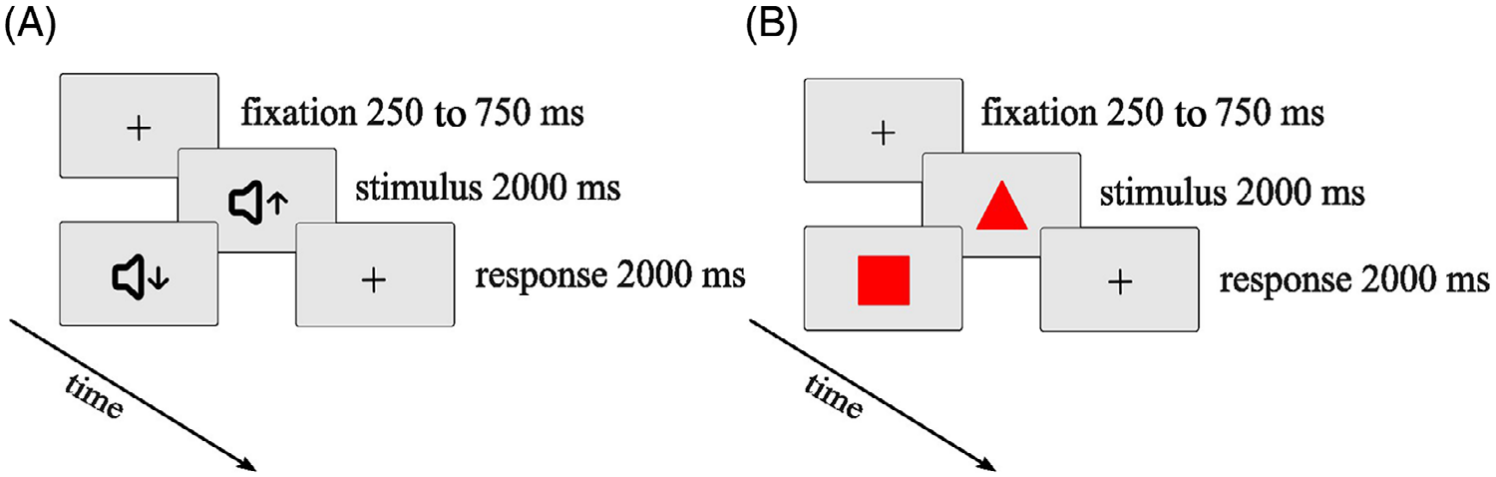
Graphical representation of trial structures and timings for both types of oddball tasks. (A) Auditory oddball task: Participants were presented with either the oddball or the standard stimulus (high or low frequency tone, respectively). (B) Visual oddball task: Participants were presented with either the oddball or standard stimulus (triangle or square, respectively).

*Oddball task*. The task was programmed using MATLAB R2015a (version 8.5.0.197613, MathWorks, Inc, 2015) and Cogent 2000 software. It was presented on a 48.26 cm (19‐inch) computer screen with a resolution of 1024 × 768 pixels. Participants were seated at a distance of 80 cm from the screen, with their head stabilized by a chin rest and head rest mounted on the desktop. All stimuli, including background and fixation crosses, were luminance adjusted to 50% brightness to control for luminance‐related effects on PD. For the auditory oddball task, the stimulus was presented binaurally through headphones while participants were fixating on a fixation cross in front of a checkered background. The oddball was a high‐frequency tone (2000 Hz) and the standard stimulus a low‐frequency tone (1000 Hz), the loudness of both was adapted to the individual's hearing ability prior to the task (Figure 1A). For the visual oddball task, the oddball stimulus was a triangle and the standard stimulus was a square, both filled with a red color and outlined with a dark border. Stimuli were presented in front of a gray checkered background (Figure 1B). Responses to the oddball task were given using a specific button on a four‐alternative button box. Recordings in the LC in animal studies suggest that the LC is also active during response initiation.^59^ To maximize PD to oddball stimuli, participants were therefore asked to only respond to oddball stimuli (cf. eight for a similar approach in a study recording LC responses during an oddball task). For auditory and visual oddball tasks, a trial began with a jittered fixation cross (250 to 750 ms, mean duration 500 ms), followed by a visual or auditory stimulus that lasted 2000 ms, followed by a response period of 2000 ms. Participants were instructed to let their index finger rest on the button after the practice to avoid pressing the wrong button.

The oddball task consisted of eight alternating blocks, beginning with the visual oddball task and then alternating between the task types. Participants were reminded of the task instructions at the beginning of each block. Each block consisted of five oddball trials and 20 standard trials, resulting in a total number of 20 oddball and 80 standard trials per task type. Participants were asked to respond to the oddball stimuli only. All participants completed a short practice session for both stimulus types.

**FIGURE 2:**
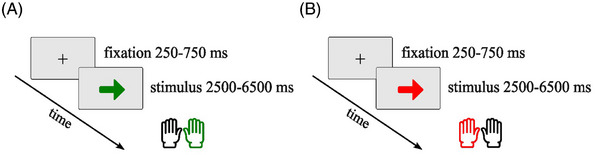
Graphical representation of trial structures and timings in Simon task. (A) Congruent trials. For the green arrow, participants had to respond with the hand that was congruent with the arrow direction. (B) Incongruent trials. For the red arrow, participants had to respond with the hand that was incongruent with the arrow direction.

*Simon task*. The task was performed in a MRI scanner and presented on a 48.26‐cm (19‐inch) computer screen with a resolution of 1024 × 768 pixels, viewed through a mirror attached to the head coil. Stimuli were solid red or green arrows presented in the center of the screen, preceded by a gray fixation cross. As for the oddball task, checkered background, fixation cross, and stimuli were luminance controlled to 50% brightness. A trial began with a jittered fixation cross (250 to 750 ms, mean duration 500 ms) followed by an arrow stimulus for 2500 or 4500 ms (based on average RTs during practice). A response was counted if it was executed during stimulus presentation. MR‐compatible button boxes in each hand were used for response execution with left and right index fingers. YAs (*n* = 22) and OAs (*n* = 22) had an average response deadline of 2500 ms, whereas MCI patients (*n* = 20) had an average response deadline of 3000 ms, with five patients requiring an extended deadline of 4500 ms. Participants were asked to respond differently to the green and red arrows. For green arrows (congruent condition), participants pressed the button with the hand corresponding to the direction of the arrow (i.e., a rightward‐pointing arrow required a right‐hand response) (Figure 2A). For red arrows (incongruent condition), participants pressed the button with the hand opposite to the direction of the arrow (rightward‐pointing arrow required a left‐hand response) (Figure 2B). Instructions and a practice session of 12 trials were completed outside the MRI scanner on a desktop computer. The main test session consisted of 200 trials, of which 160 trials were congruent stimuli (green arrow) and 40 were incongruent trials (red arrow). Both trial conditions consisted of an equal number of left‐pointing and right‐pointing arrows.

### 2.6 Acquisition and preprocessing of pupillometric data

PD was recorded continuously throughout the experiment and segmented in a time window of 200 ms before and 2500 ms after relevant events (i.e., oddball or target onset, onset of congruent or incongruent stimulus) for assessing condition effects in phasic PD. To enable comparisons of phasic PD regardless of individual pupil size variations and distance to the eye tracker camera, segmented pupil data were concatenated and z‐scored per individual before assessing condition effects.^60^ Phasic pupil responses were assessed relative to a 200‐ms baseline prior to stimulus presentations, that is, the mean PD in the baseline window was subtracted from the event‐related PD for each trial. Moreover, given that intraindividual stability and noise in physiological processes can be expected to be altered in aging or MCI,^61^ an indicator of measurement noise in PD was calculated based on variations in PD across time for each trial in the 200‐ms pretrial window, averaged across all trials per participant, yielding an individual‐specific measure of PD variations in the absence of task‐related events (referred to here as baseline noise).^60^ In case of group differences in baseline noise in PD recordings, estimates of baseline noise were included as covariate. Missing data due to artifacts and blinks were detected in MATLAB 2015b using the MATLAB‐based toolbox FieldTrip (http://www.fieldtriptoolbox.org/). Missing data were replaced by linear interpolation using the interp1.m implemented in MATLAB. As missing data due to blinks or artifacts are typically preceded and followed by corrupted data (e.g., due to the eyelid closing and opening), data were interpolated within time periods of 200  or 30 ms before and after large or small artifacts, respectively. Interpolated data were visually inspected and trials excluded if fewer than three trials were available in either condition (see Table S2 in the Supplement for trial numbers and group comparisons).

To compare condition effects, the mean PD for each participant was estimated by averaging the PD for the auditory oddball and Simon tasks during the 1000‐ to 2000‐ms time periods. Since the visual stimulus elicited a faster pupil response than the auditory stimulus during the oddball task, the mean PD for the visual task was computed within a time range of 500 to 1500 ms after stimulus onset.

### 2.7 Structural LC imaging

Scanning was performed with a 3T Siemens TIM Trio System and a 32‐channel head coil. LC volumes were assessed using high‐resolution (0.6×0.6×3mm, 20 slices) magnetization transfer‐weighted (MT‐weighted) 3D Gradient Echo images (FLASH), (repetition time = 24.50 ms, echo time = 3.35 to 11.75 ms, FA = 12), averaged across three repetitions. Quality assurance checks were performed to identify image artifacts, and sequences were repeated when recording artifacts were detected. Likely due to neuromelanin deposits in the LC, the LC is visible as a bright structure in so‐called neuromelanin‐sensitive scans.^62^, ^63^ As deposits are accumulating within LC cells, LC brightness is therefore interpreted as an indirect indicator of the structural integrity of the LC.^64^ The LC mask was determined using the consensus between two independent raters' voxel‐wise segmentations in ITK‐SNAP.^65^ To calculate a measure of LC integrity, neuromelanin signal intensity was taken as a ratio score of signal intensity in the LC mask to signal intensity in a reference region without neuromelanin located nearby in the pons. LC contrast ratio (CR) was then computed by dividing the difference between the intensity of the LC signal and the reference signal by the reference signal intensity.^66^ The size and location of the reference region used was determined based on previous studies.^67^ Two independent raters (DH and MD) manually segmented the LC using ITK‐SNAP, as previously described.^43^ LC integrity was calculated as the ratio score of signal intensity in the conjunction mask across both raters relative to the signal intensity in a reference region located in the pons not assumed to contain neuromelanin.^42^, ^43^ Sørensen–Dice coefficients for inter‐rater consistency across the three groups were 0.70 (YAs), 0.65 (OAs), and 0.57 (MCI), respectively. Signal brightness in the three brightest voxels was averaged per participant and used as a more robust measure of LC integrity^68^.

### 2.8 Data analyses

Data quality control involved several steps. First, participants were excluded if they had missing pupil data, had insufficient behavioral data, or failed to perform the task according to task instructions. Specifically, RTs smaller than 200 ms were considered implausibly fast and excluded from the analysis. RTs greater or smaller than three standard deviations from the group mean were considered potential outliers and removed. Given the right‐skewed distribution, RTs were log‐transformed for normalization. Finally, each participant's aggregated PD data were visually inspected to identify subjects with consistent measurement abnormalities or signal instability across multiple trials.

Missing data on neuropsychological tests and LC integrity measures were imputed using the expectation‐maximization algorithm, using age as a predictor. In total, out of the 93 participants, four (all MCI patients) for ACE‐R total, four (all MCI patients) for ACE‐R memory, four (all MCI patients) for ACE‐R attention/orientation, four (all MCI patients) for ACE‐R verbal fluency, four (all MCI patients) for ACE‐R language, four (all MCI patients) for ACE‐R visuospatial, two (two MCI patients) for MMSE, and three subjects (one YAs and two MCI) were imputed for LC integrity. For the ACE‐R assessment, ACE‐R total showed means of 94.43 (SD = 4.75) for YAs, 96.03 (SD = 3.37) for OAs, and 78.45 (SD = 11.70) for MCI patients. ACE‐R memory subscale means were 24.27 (SD = 2.11) for YAs, 24.63 (SD = 2.00) for OAs, and 15.79 (SD = 6.44) for MCI patients. ACE‐R attention/orientation subscale showed means of 17.87 (SD = 0.43) for YAs, 17.80 (SD = 0.48) for OAs, and 15.21 (SD = 2.02) for MCI patients. ACE‐R verbal fluency subscale means were 12.57 (SD = 1.43) for YAs, 12.93 (SD = 1.14) for OAs, and 9.34 (SD = 2.80) for MCI patients. ACE‐R language subscale means were 24.17 (SD = 2.15) for YAs, 25.53 (SD = 0.77) for OAs, and 24.03 (SD = 2.38) for MCI patients. ACE‐R visuospatial ability subscale means were 15.43 (SD = 1.13) for YAs, 15.20 (SD = 1.09) for OAs, and 14.07 (SD = 2.72) for MCI patients. Average MMSE scores were 29.53 (SD = 1.07) for YAs, 29.43 (SD = 0.72) for OAs, and 25.19 (SD = 2.79) for MCI patients. LC integrity measurements prior to imputation showed means of 0.13 (SD = 0.03) for YAs, 0.16 (SD = .04) for OAs, and 0.10 (SD = 0.03) for MCI patients. The imputation procedure did not significantly alter the distribution of these initial values (see Table S1 in the Supplement for detailed statistics with imputed values).

Statistical analyses were performed with R version 4.4.1 (2024‐06‐14).^69^ The following R packages were used: ggplot2 (3.5.1) for data visualization, car (3.1.3) for Levene's test, afex (1.4.1) for ANOVAs, emmeans (1.10.4) for estimated marginals means and pairwise comparisons among the means, effectsize (0.8.9) for calculating effect sizes for *t*‐tests, cocor (1.1.4) for Williams' *t*‐test and Fisher's *z*‐test. Significance levels were set at α = 0.05. Multiple pairwise comparisons were conducted using estimated marginal means (emmeans package) and adjusted using multivariate *t*‐distribution correction to control family‐wise error rate. Effect sizes were calculated using partial eta squared (η^2^p). For one‐way ANOVAs, eta squared (η^2^) was used. Cohen's *d* (*d*) was calculated to assess effect size for independent *t*‐tests.

### 2.9 Statistical analyses

To verify age matching between the OAs and MCI patients, we performed a two‐sample *t*‐test, which confirmed no significant age differences between the groups (see “Methods,” Section 2.2, for a description of participants).

For PD on the oddball tasks, we performed a repeated measures ANOVA with two within‐subjects factors: stimulus type (two levels: standard, oddball) and task (two levels: auditory, visual) and a between‐subjects factor: group (two levels: OAs, MCI). For PD within each task, we performed a repeated measures ANOVA with a within‐subjects factor: stimulus type (two levels: standard, oddball) and a between‐subjects factor: group (two levels: OAs, MCI). Baseline pupillary noise as defined by temporal variations within the baseline window differed significantly between groups, *t*(35) = −2.02, *p* = 0.05, *d* = 0.68, with OAs showing lower baseline noise (M = 0.14) compared to MCI patients (M = 0.16). Given these group differences, we mean‐centered and included baseline noise as a covariate in subsequent analyses. Levene's test indicated homogeneity of variances across groups for baseline noise (*F*[1, 35] = 2.76, *p* = 0.10), mean PD for standard stimuli in the visual oddball task (*F*[1, 35] = 0.03, *p* = 0.86), mean PD for oddball stimuli in the auditory oddball task (*F*[1, 35] = 1.69, *p* = 0.20), and mean PD for standard stimuli in the auditory oddball task (*F*[1, 35] = 0.13, *p* = 0.71). However, this assumption was violated for mean PD for oddball stimuli in the visual oddball task (*F*[1, 35] = 5.15, *p* = 0.02). For behavioral assessments, we used a repeated measures ANOVA with a within‐subjects factor: task (two levels: auditory, visual) and a between‐subjects factor: group (two levels: OAs, MCI). In addition, we used two‐sample *t*‐tests to examine differences between the OAs and MCI groups in performance within each oddball task. Levene's test indicated homogeneity of variances across groups for hit rate in the visual oddball task (*F*[1, 35] = 0.95, *p* = 0.33), discrimination accuracy in the visual oddball task (*F*[1, 35] = 1.52, *p* = 0.22), RTs in the visual oddball task (*F*[1, 35] = 0.05, *p* = 0.82), and RTs in the auditory oddball task (*F*[1, 35] = 0, *p* = 0.99). However, this assumption was violated for the hit rate in the auditory oddball task (*F*[1, 35] = 10.46, *p* < 0.01) and the discrimination accuracy in the auditory oddball task (*F*[1, 35] = 7.03, *p* = 0.01). In this case, the Welch two‐sample *t*‐test was used.

For PD on the Simon task, we used a repeated measures ANOVA with a within‐subjects factor: condition (two levels: congruent, incongruent) and a between‐subjects factor: group (three levels: YAs, OAs, and MCI). As in the oddball tasks, analysis of baseline pupil noise revealed significant group differences (*F*[1, 59] = 14.74, *p* < 0.001, η^2^p = 0.33). *Post hoc* comparisons revealed that YAs had lower baseline noise (M = 0.16) compared to both OAs (M = 0.19) and MCI patients (M = 0.22), while OAs also had significantly lower baseline noise compared to MCI patients. Therefore, baseline noise per individual was mean‐centered and included as a covariate in the analysis of the pupil data. Levene's test indicated homogeneity of variances across groups for baseline noise (*F*[2, 59] = 2.61, *p* = 0.08), mean PD during congruent trials (*F*[2, 59] = 0.06, *p* = 0.93), and mean PD during the incongruent trials (*F*[2, 59] = 0.22, *p* = 0.80). For behavioral assessments, we performed a repeated measures ANOVA with a within‐subjects factor: condition (two levels: congruent, incongruent) and a between‐subjects factor: group (three levels: YAs, OAs, and MCI). Levene's test indicated homogeneity of variances across groups for RTs during correctly performed incongruent trials (*F*[2, 59] = 2.94, *p* = 0.06). However, this assumption was violated for accuracy during correctly performed congruent trials (*F*[2, 59] = 6.45, *p* < 0.01) and correctly performed incongruent trials (*F*[2, 59] = 4.54, *p* = 0.01), and for RTs during correctly performed congruent trials (*F*[2, 59] = 4.22, *p* = 0.01) and incorrectly performed incongruent trials (*F*[2, 37] = 7.40, *p* <0 .01).

We examined relationships between variables using Spearman's correlations, analyzing associations between (1) PD and behavioral performance, (2) PD and LC integrity, and (3) task measures and cognitive screening measures. First, we conducted correlational analyses between the variables of interest. For those correlations that showed significant relationships, we performed outlier analyses using Cook's distance to identify influential data points (outliers). Observations were considered highly influential and eliminated from the correlational analyses if their Cook's distance exceeded 4/*n* (where *n* is the sample size of each group). For the single oddball task correlational analysis, an average of one case of YAs, one case of OAs, and two cases of MCI patients were excluded after being identified as highly influential outliers. For the Simon task correlational analyses, an average of 0.66 YAs, 0.33 OAs, and 0.66 MCI patients were excluded from each group across the six correlational tests. For the single correlational analysis of incorrectly performed incongruent trials in the Simon task, two participants were excluded: one from the OAs and one from the MCI patients. While Williams' *t*‐test was used to assess significantly different correlations within the same group, Fisher's *z*‐test was used to compare the difference between two correlations in two independent groups.

To identify relationships across individuals in the context of small sample sizes, we performed a multiple linear regression analysis with an interaction term to examine the relationship between PD for congruent/incongruent trials and oddball/standard stimuli and LC integrity as well as RTs for congruent/incongruent trials and oddball/standard stimuli and LC integrity. Our analysis focused only on older groups (OAs and MCI patients), as LC integrity in younger age groups is likely more dominated by interindividual differences in neuromelanin accumulation rather than cell loss.^70^ A binary group variable was created, where 0 represented OAs and 1 represented MCI patients. The linear model was constructed with mean PD as the dependent variable and group and LC integrity as the independent variables. Additionally, the linear models for the RTs were constructed with RTs as the dependent variable and group and LC integrity as the independent variables. The analysis was performed using the lm() function in R.

## 3 RESULTS

### 3.1 Oddball task

#### 3.1.1 Behavioral results

**FIGURE 3:**
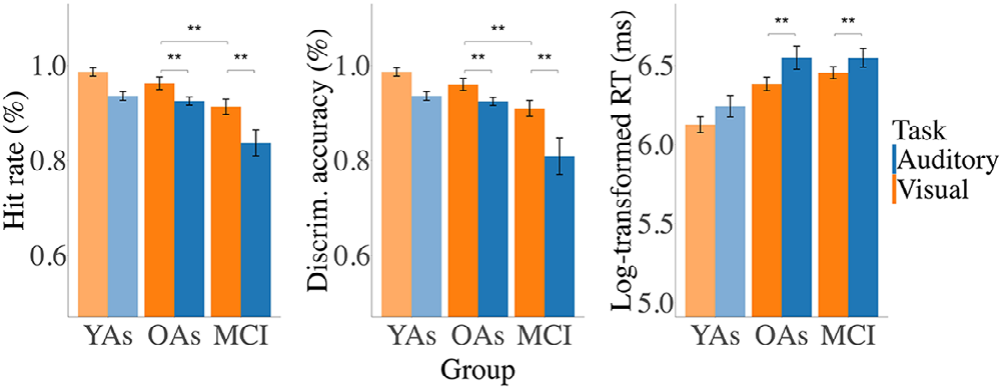
Mean percentage of hit rate, discrimination accuracy, and RTs across two types of oddball tasks. Note that due to small sample sizes, data from young adults is not included in statistical analyses and included for illustrative purposes only (indicated by lighter shading). Error bars represent ± 1 standard error. Significant differences are indicated by asterisks (**p < 0.01).

*Between tasks (visual oddball vs auditory oddball)*. A repeated measures ANOVA (group × task) for accuracy revealed a group effect, with OAs showing higher accuracy than patients with MCI regardless of the stimulus modality, with a higher hit rate (OAs: M = 0.94; MCI: M = 0.87) (*F*[1, 35] = 8.38, *p* < 0.01, η^2^p = 0.19) and discrimination accuracy (hit rate minus false alarm rate) (OAs: M = 0.94; MCI: M = 0.85) (*F*[1, 35] = 7.46, *p* = 0.01, η^2^p = 0.17). In addition, our analysis revealed a main effect of task, indicating that participants performed better on the visual compared to the auditory oddball task, in terms of faster RTs (visual: M = 6.42; auditory: M = 6.55) (*F*[1, 35] = 10.31, *p* < 0.01, η^2^p = 0.23), higher hit rates (visual: M = 0.93; auditory: M = 0.88) (*F*[1, 35] = 9.45, *p* < 0.01, η^2^p = 0.21), and higher discrimination accuracy (visual: M = 0.93; auditory: M = 0.86) (*F*[1, 35] = 7.54, *p* < 0.01, η^2^p = 0.17). No interaction effects between group and task were observed. A repeated measures ANOVA (group × task) for RTs did not reveal significant differences between groups, suggesting that both groups took the same time responding to oddballs, while processing accuracy was higher in OAs (Figure 3).

*Within tasks*. A *t*‐test for hit rate revealed significantly better performance in OAs compared to MCI participants in the auditory oddball task, *t*(26.24) = 3.07, *p* < 0.01, *d* = 0.83, OAs (M = 0.92), and MCI (M = 0.83), as well as in the visual oddball task, *t*(35) = 2.14, *p* = 0.03, *d* = 0.72, OAs (M = 0.96), and MCI (M = 0.91). Similarly, a *t*‐test for discrimination accuracy was significantly higher in OAs than in MCI in the auditory oddball task, *t*(24.12) = 2.91, *p* < 0.01, *d* = .77, OAs (M = 0.92), and MCI (M = 0.80) as well as in the visual oddball task, *t*(35) = 2.11, *p* = 0.04, *d *= 0.71, OAs (M = 0.96) and MCI (M = 0.90). A *t*‐test on RTs for hits revealed no difference between groups in the auditory and visual oddball tasks.

#### 3.1.2 Pupillometry results

**FIGURE 4:**
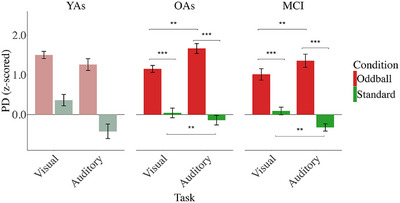
Mean pupil dilation across visual and auditory oddball tasks, assessed as mean in the time window (from 1 to 2 s and from 0.5 to 1.5 s after stimulus onset in auditory oddball and visual oddball tasks, respectively). Note that due to small sample sizes, data from young adults is not included in statistical analyses and included for illustrative purposes only (indicated by lighter shading). Error bars represent ± 1 standard error. Significant differences are indicated by asterisks (***p < 0.001, *p < 0.05).

**FIGURE 5:**
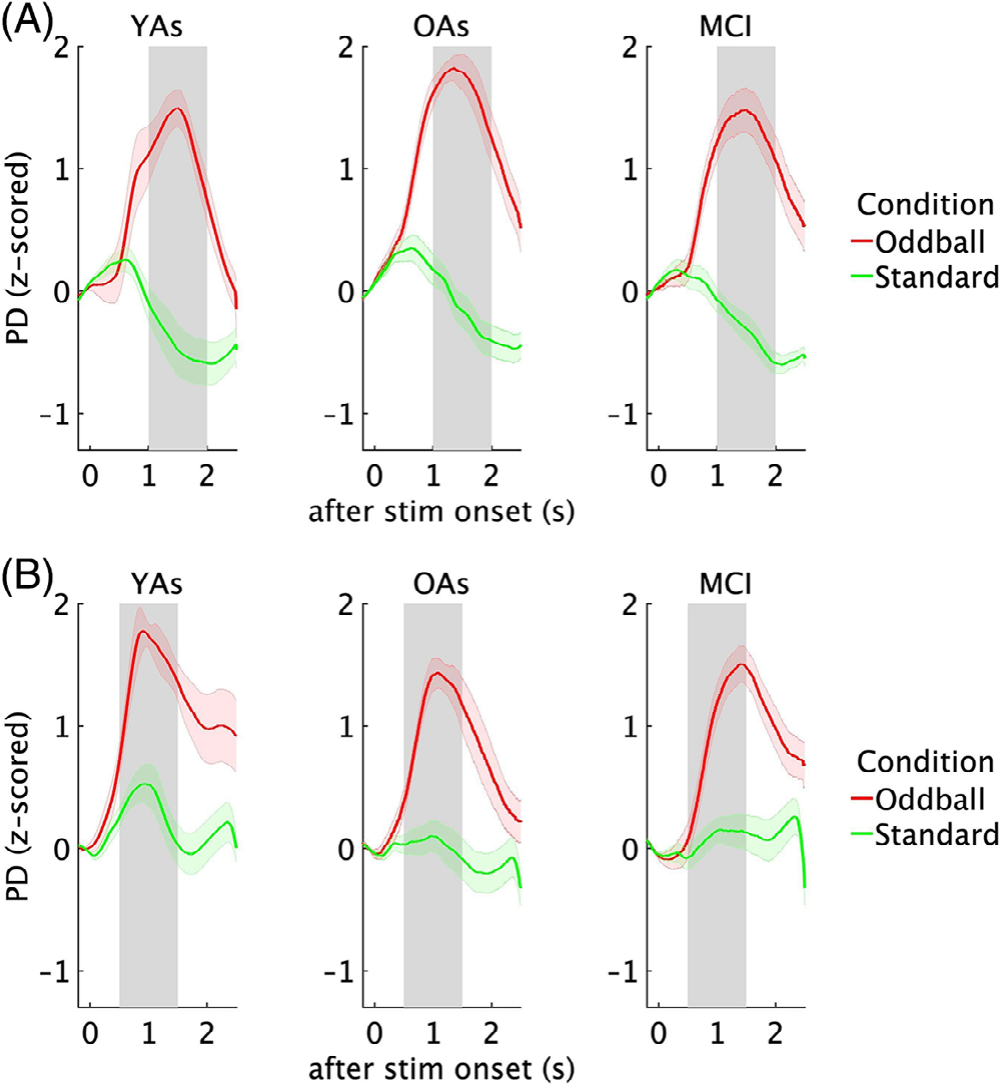
PD change over time in oddball task. (A) Pupil dilation (PD) change over time from 1 to 2 s after stimulus onset for oddball and standard auditory oddball stimuli. (B) PD change over time from 0.5 to 1.5 s after stimulus onset for oddball and standard visual oddball stimuli.

*Between tasks*. A repeated measures ANOVA (group × condition × task) for PD revealed the expected main effect of greater PD for oddball (M = 1.28) compared to standard stimuli (M = −0.07) (*F*[1, 34] = 195.59, *p* < 0.001, η2p = 0.85) as well as a task × condition interaction effect (*F*[1, 34] = 27.24, *p* < 0.001, η^2^p = 0.44) (Figure 4), showing that PD was greater for oddball than standard stimuli in both visual (oddball: M = 1.07, standard: M = 0.07) (*p* < 0.001) and auditory task (oddball: M = 1.49, standard: M = −0.22) (*p* < 0.001). For oddball stimuli, PD was higher in the auditory than the visual task (*p* < 0.01). For standard stimuli, higher PD was in the visual compared to auditory task (*p* < 0.01). Moreover, we found that the difference between PD for oddball and standard stimuli was greater on the auditory than the visual task (*p* < 0.001). No interaction between group and condition was found, indicating comparable pupil responses to different stimulus types for OAs and patients with MCI. No other interactions were observed. For the change in PD over time across the different conditions in the oddball task, see Figure 5.

*Within tasks*. A repeated measures ANOVA (group × condition), analyzed separately for the visual and auditory oddball tasks, indicated a higher PD response for oddball stimuli (visual: M = 1.07; auditory: M = 1.49) compared to standard stimuli (visual: M = 0.07; auditory: M = −0.22) in both the auditory oddball task (*F*[1, 34] = 176.10, *p* < 0.001, η^2^p = 0.83) and the visual oddball task (*F*[1, 34] = 85.41, *p* < 0.001, η^2^p = 0.71). Again, there were no main effect of the group and interactions between group and condition as well as between the baseline pupillary noise covariate and condition.

#### 3.1.3 Correlations between oddball task performance, pupil responses, cognitive screening measures, and LC integrity

A significant correlation was observed between greater PD for the oddball stimuli and faster RTs for hits in both OAs, *r* = −0.81, *p* < 0.01, and MCI patients, *r* = −0.57, *p* < 0.01, on the visual task (Figure S6 in the Supplement). This relationship was not apparent for standard stimuli in OAs, *r* = −0.08, *p* = 0.78, or MCI patients, *r* = −0.30, *p* = 0.18 (Figure S7 in the Supplement), suggesting that participants who can devote more attention and effort to the task‐relevant stimuli may respond faster. A Williams' *t*‐test revealed that the correlation between PD and RTs was significantly stronger for PD for oddball stimuli (*r* = −0.81) compared to PD for standard stimuli (*r* = −0.08) in OAs, *t*(10) = −3.65, *p* < 0.01. However, although the correlation appeared to be stronger for oddball stimuli (*r* = −0.57) compared to standard stimuli (*r* = −0.30), in MCI patients, this difference did not reach statistical significance, *t*(18) = −1.08, *p* = 0.28. Because correlational coefficients for subsequent Williams' *t*‐tests should be calculated from the same sample, these correlational analyses were performed on data from the exact same individuals within each group (OAs: *n* = 13, MCI: *n* = 21). Furthermore, Fisher's test showed that the association between PD for standard stimuli and RTs was not substantially stronger in OAs than in MCI patients, *z* = 0.57, *p* = 0.56. This pattern was not replicated in the auditory oddball task, as no significant correlations were observed between PD and behavioral data. In addition, attention scores from the ACE‐R subtest did not correlate significantly with either PD or task performance in the oddball tasks. There were no significant correlations between LC integrity and PD in OAs and MCI patients.

### 3.2 Simon task

#### 3.2.1 Behavioral results

**FIGURE 6:**
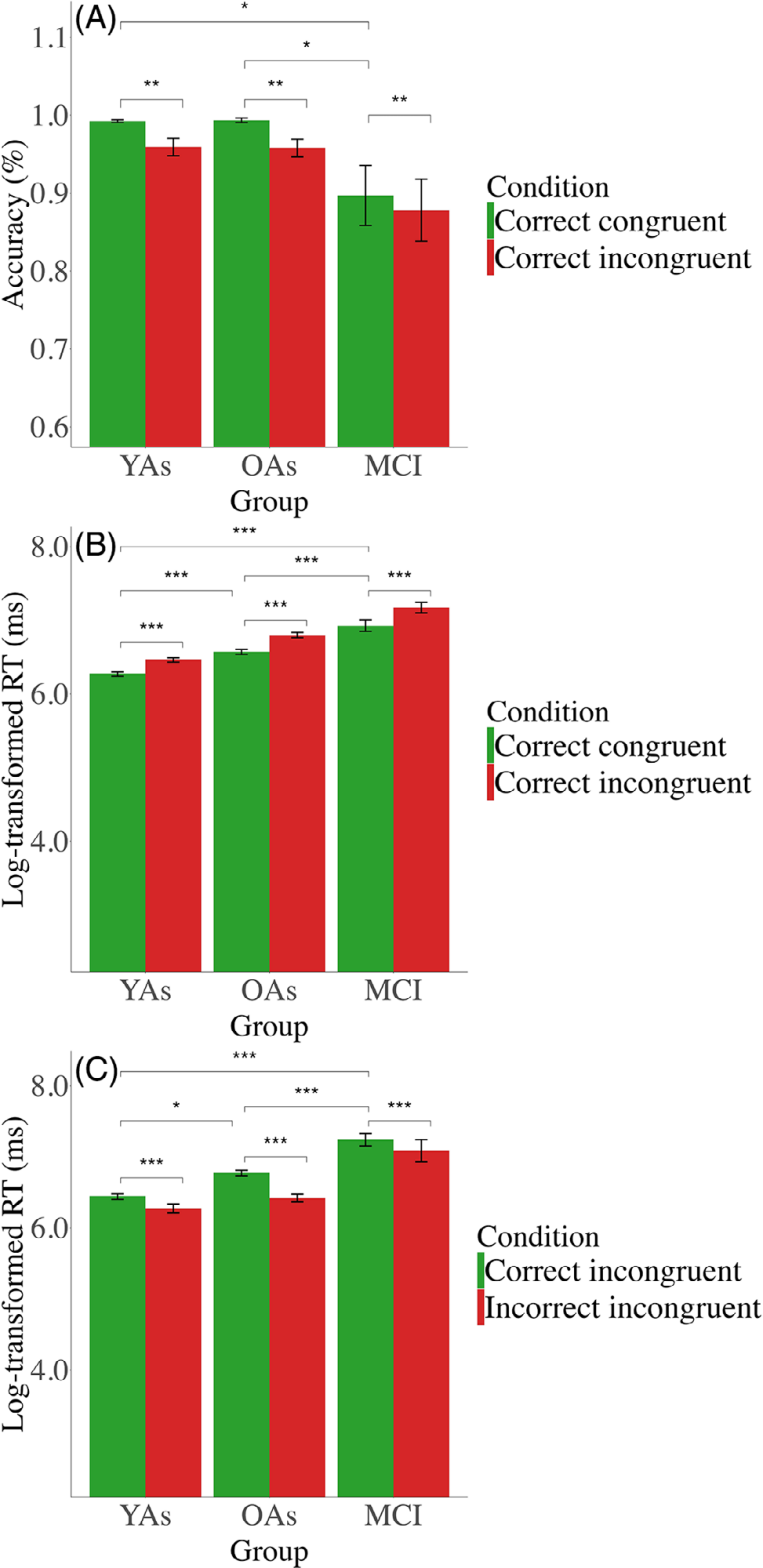
Behavioral performance in Simon task. Error bars represent ± 1 standard error. Significant differences are indicated by asterisks (***p < 0.001, **p < 0.01, *p < 0.05). (A) Mean percentage of correct congruent and correct incongruent trials. (B) Mean reaction times (RTs) in correct congruent and correct incongruent trials. (C) Mean RTs in correct incongruent and incorrect incongruent trials.

As YAs were fully included in assessments of the Simon task, statistical analyses include all three groups. A repeated measures ANOVA (group × condition) for accuracy revealed the expected main effect of higher accuracy for congruent trials (M = 0.96) as compared to incongruent trials (M = 0.93) (*F*[1, 59] = 10.42, *p* < 0.01, η^2^p = 0.15) and a group main effect (*F*[2, 59] = 5.52, *p* < 0.01, η^2^p = 0.16), with both YAs (M = 0.97) and OAs (M = 0.97) showing better accuracy than patients with MCI (M = 0.88; YAs vs MCI: *p* = 0.01; OAs vs MCI: *p* = 0.01), but no difference between YAs and OAs (*p* = 01.00). No significant group × condition interaction effect was observed (Figure 6A). A further one‐way ANOVA for accuracy revealed a group effect for congruent trials (*F*[2, 59] = 6.53, *p* < 0.01, η^2^ = 0.18), showing both YAs (M = 0.99) and OAs (M = 0.99) had significantly better accuracy than MCI patients (M = 0.89; YAs vs MCI: *p* < 0.01; OAs vs MCI: < 0.01), with no difference between YAs and OAs (*p* = 0.99). For incongruent trials, a one‐way ANOVA also showed a group effect (*F*[2, 59] = 3.63, *p* = 0.03, η^2^ = 0.11). However, pairwise comparisons showed no significant differences between groups (YAs vs OAs: *p* = 0.99, OAs vs MCI: *p* = 0.06), and the difference between YAs and MCI patients did not reach statistical significance (*p* = 0.052).

A repeated measures ANOVA (group × condition) for RTs on correct congruent versus correct incongruent trials showed the expected main effect of condition (*F*[1, 59] = 313.04, *p* < 0.001, η^2^p = 0.84) and group (*F*[2, 59] = 50.00, *p* < 0.001, η^2^p = 0.63) (Figure 6B), with RTs being faster for correct congruent trials (M = 6.59) compared to correct incongruent trials (M = 6.81). The pattern of faster RTs during correct congruent trials is in line with enhanced cognitive control requirements on incongruent trials. Regarding group differences, MCI patients had slower RTs (M = 7.05) than both OAs (M = 6.68, *p* < 0.001) and YAs (M = 6.37, *p* < 0.001), and the latter two groups also showed significant differences between each other (*p* < 0.001). There was no significant group × stimulus type interaction effect for RTs on correct congruent versus correct incongruent trials. A one‐way ANOVA for RTs found a group effect for congruent trials (*F*[2, 59] = 42.21, *p* < 0.001, η^2^ = 0.59), with MCI being the slowest (M = 6.93) compared to both YAs (M = 6.27, *p* < 0.001) and OAs (M = 6.57, *p* < 0.001), while OAs were slower on correct congruent trials compared to YAs (*p* < 0.001). For incongruent trials, a one‐way ANOVA indicated a group effect (*F*[2, 59] = 53.75, *p* < 0.001, η^2^ = 0.64), with MCI being the slowest (M = 7.17) compared to both YAs (M = 6.46, *p* < 0.001) and OAs (M = 6.80, *p* < 0.001), and OAs were slower on correct incongruent trials compared to YAs (*p* < 0.001).

A repeated measures ANOVA (group × condition) for RTs on correct incongruent versus incorrect incongruent trials revealed significant main effects of condition (*F*[1, 37] = 28.15, *p* < 0.001, η^2^p = 0.43) and group (*F*[2, 37] = 33.52, *p* < 0.001, η^2^p = 0.64) (Figure 6C). Specifically, incorrect incongruent responses (M = 6.59) were executed faster than correct incongruent responses (M = 6.82), consistent with the notion that incorrect responses often represent premature responses that are not inhibited by top‐down motor control.^63^ Patients with MCI had the slowest RTs (M = 7.16) compared to both YAs (*p* < 0.001) and OAs (*p* < 0.001), while OAs (M = 6.60) were slower than YAs (M = 6.36, *p* = 0.04). There was no significant group × stimulus type interaction effect for RTs on correct incongruent versus incorrect incongruent trials.

#### 3.2.2 Pupillometry results

**FIGURE 7:**
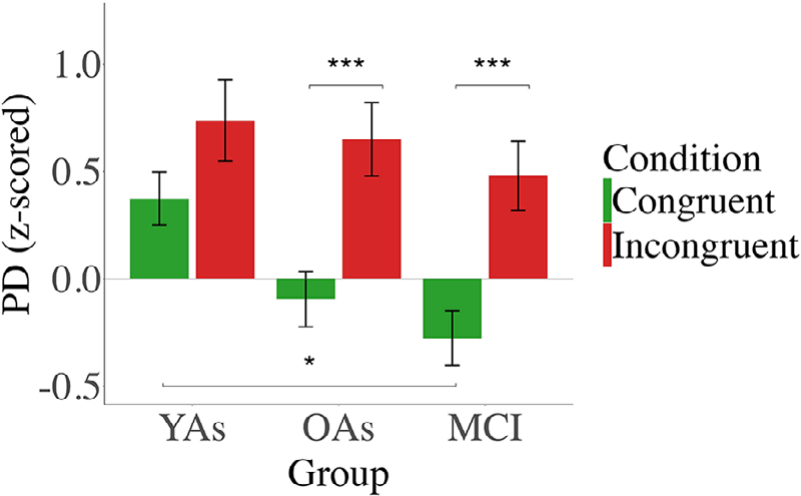
Mean pupil dilation in Simon task, assessed as mean in time window (from 1 to 2 s after stimulus onset). Error bars represent ± 1 standard error. Significant differences are indicated by asterisks (***p < 0.001, *p < 0.05).

**FIGURE 8:**
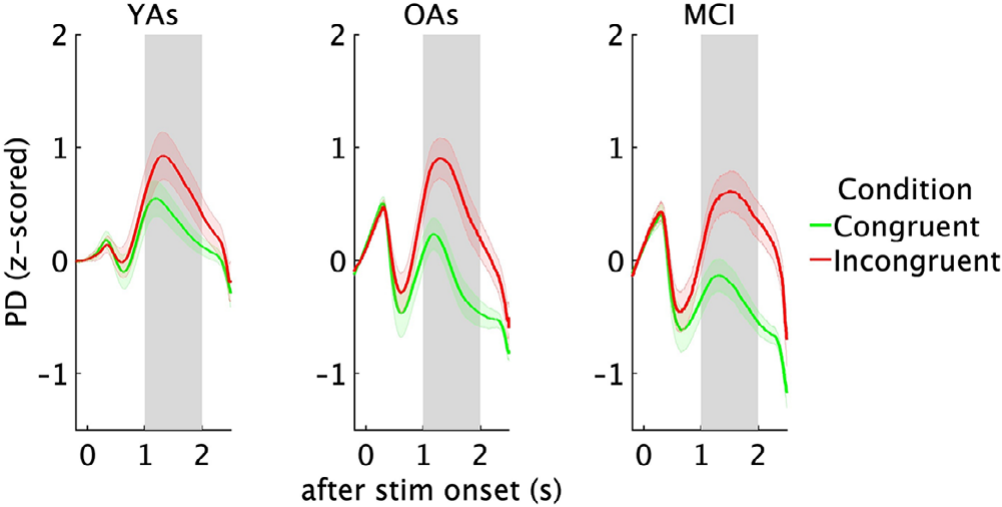
Pupil dilation change over time from 1 to 2 s after stimulus onset for congruent and incongruent Simon task stimuli.

The repeated measures ANOVA (group × condition) for PD showed the expected main effect of condition (*F*[1, 58] = 73.47, *p* < 0.001, η2p = 0.55), with higher PD for incongruent (M = 0.62) versus congruent trials (M = 0.002), suggesting more attention and/or effort when processing incongruent stimuli. A condition × group interaction (*F*[2, 58] = 3.22, *p* = 0.04, η2p = 0.10) indicated greater PD for incongruent (M = 0.65) versus congruent trials (M = −0.09) in OAs (*p* < 0.001) and for incongruent (M = 0.55) versus congruent (M = −0.25) trials in MCI patients (*p* < 0.001), whereas YAs somewhat unexpectedly did not show a significant difference between congruent (M = 0.34) and incongruent conditions (M = 0.67) (Figure 7). Additionally, PD was found to be greater for congruent trials in YAs than in MCI patients (*p* = 0.03). No significant interaction was found between the baseline pupillary noise covariate and condition. For the change in PD over time across the different conditions in the Simon task, see Figure 8.

#### 3.2.3 Correlations between oddball task performance, pupil responses, cognitive screening measures as well as LC integrity

Examining the correlations between behavioral data and PD revealed faster RTs for correctly performed incongruent trials in YAs who showed a greater PD on incongruent trials, *r* = −0.63, *p* < 0.01 (Figure S8 in the Supplement), suggesting that YAs who expended more cognitive control responded faster. Second, faster RTs on incorrectly performed incongruent trials were associated with increased PD on incongruent trials in YAs, *r* = −0.61, *p* = 0.02 (Figure S9 in the Supplement). Increased PD on erroneous incongruent trials might reflect increased (albeit unsuccessful) efforts on incongruent trials or heightened attentional arousal in the context of a typically conscious action slip error.^71^ The same was observed for MCI patients, *r* = −0.82, *p* < 0.01 (Figure S9 in the Supplement). In addition, we found that a more pronounced Simon effect (i.e., PD incongruent minus PD congruent) correlated with a faster RTs on correctly performed incongruent trials in YAs, *r* = −0.62, *p* < 0.01 (Figure S10 in the Supplement).

To determine whether interindividual differences in attention and memory capacity contributed to interindividual differences in the Simon task, we correlated attention and memory scores from the ACE‐R subtests with PD and behavioral data from the Simon task. We found that individuals with higher memory capacity showed faster RTs for correct congruent trials in MCI patients, *r* = −0.52, *p* = 0.02 (Figure S11 in the Supplement). Similarly, higher memory scores were correlated with better performance on correct congruent, *r* = 0.64, *p* < 0.01 (Figure S12 in the Supplement) and incongruent trials in the MCI group, *r* = 0.46, *p* = 0.03 (Figure S13 in the Supplement). Similar results were observed for the MMSE: higher memory scores were associated with a higher percentage of correctly completed congruent trials in the MCI group, *r* = 0.51, *p* = 0.02 (Figure S14 in the Supplement). Linear regression analyses did not reveal significant associations between LC integrity and PD in the Simon task in OAs and MCI patients. However, we found that in MCI patients, higher LC integrity was associated with faster RTs for correct incongruent trials (*b* = −5.26, *p* = 0.01) (Figure S15 in the Supplement).

## 4 DISCUSSION

The results of this study showed that the expected involvement of PD in top‐down attentional modulation or cognitive effort during the oddball and Simon tasks occurred to a similar extent in both elderly groups, i.e., healthy OAs and MCI patients. For the oddball task, the oddball effect, represented by increased PD for oddball stimuli, was replicated in OAs and MCI patients. Performance was better in OAs than in MCI patients, as indicated by a higher hit rate for oddball stimuli and discrimination accuracy in OAs compared to MCI patients. For the Simon task, PD was increased for incongruent as compared to congruent trials in OAs and MCI patients, but not in YAs. A classic Simon effect was observed, with incongruent trials eliciting lower accuracy and longer RTs, and MCI patients performing more slowly and less accurately than OAs. Higher PD responses were associated with faster RTs in all tasks. Simon's task performance in MCI patients was linked to memory scores on the ACE‐R and MMSE.

The behavioral results of the oddball task revealed a mixed pattern of attentional resource allocation in MCI. MCI patients showed intact motor response speed with comparable RTs to healthy OAs in both oddball tasks. However, their ability to sustain attention and suppress irrelevant task stimuli is impaired, with lower accuracy levels, consistent with studies suggesting that executive function deficits are a significant predictor of the transition from MCI to AD.^72^ Importantly, OAs and MCI patients showed greater PD to oddball stimuli compared to standard stimuli. This PD‐based oddball effect is well established in auditory and visual modalities.^8^, ^73^ Although we expected a reduced attentional allocation in MCI versus healthy aging, represented by larger PD in OAs, we did not observe any group differences in different conditions. This finding suggests that MCI patients mobilize similar levels of attentional effort as healthy OAs when processing salient stimuli, despite achieving lower behavioral accuracy. Furthermore, although the results must be treated with caution given the limited sample size (Figures S1–S3 in the Supplement) in both groups, individuals with greater PD to oddball stimuli reacted faster to those stimuli, supporting PD as a marker of attentional allocation and motor effort.^2^, ^50^ Conversely, a lack of correlation with standard stimuli may suggest reduced processing resource needs for non‐salient events. Together, these findings suggest that, although MCI patients exhibit the expected behavioral decline in attentional modulation, they exert similar levels of effort to achieve these results. This dissociation between effort and behavioral outcome underscores that PD can be an informative marker for distinguishing between deficits arising from reduced attentional capacity and deficits arising from impaired response execution, slowed processing, or other contributing factors in both healthy OAs and MCI patients.

PD responses to oddball stimuli were unexpectedly larger in the auditory than in the visual task, likely reflecting greater task difficulty since both groups performed better on visual oddballs. A possible explanation is that age‐related visual acuity decline was better regulated (by receiving visual aids if needed), whereas no formal hearing assessments were conducted, despite volume adjustments being available. Interindividual differences in stimulus processing likely contributed to the variance in executive processes reflected in PD, potentially masking the relationship between greater PD and faster oddball RTs in the auditory task. While both tasks effectively identified behavioral and PD‐related executive processes in MCI, future studies should control for sensory stimulus accessibility to improve the cognitive characterization of MCI by PD.

Both oddball and Simon task stimuli involve higher attentional demands and motor execution effort, but the Simon task adds additional demand for executive control.^39^ In this sense, comparing these task types in the same population allowed us to assess whether both attentional and executive control can be examined using pupillometry in MCI. Our study replicated the Simon effect,^74^ showing reduced accuracy and slower RTs during incongruent trials. Faster RTs on incorrect incongruent trials support the idea that conflict errors represent so‐called action slips.^71^ While behavioral effects were similar across groups, MCI patients showed slower RTs specifically on incongruent trials than YAs and OAs but maintained accuracy, suggesting they exert greater cognitive effort to preserve performance. Yet, they were slower and less accurate during easier congruent trials, indicating inconsistent attention. OAs matched YAs in accuracy but exhibited slower RTs, reflecting age‐related processing slowdown with intact resource allocation.^75^

As expected, PD was increased for incongruent trials than for congruent trials in all groups, suggesting greater top‐down attentional control and cognitive effort invested in the incongruent condition.^50^ OAs and MCI patients showed enhanced PD responses for incongruent trials, which suggests that the capacity to recruit cognitive control in MCI patients is preserved. YAs showed no PD difference between trial types, which was unexpected given their presumed ability to allocate effort and attention selectively in response to increased task demands.^7^ Inspecting individual response patterns (Figure S4 in the Supplement) revealed that most YAs (15 out of 22) showed the expected larger PD for incongruent than for congruent trials. Studies with larger sample sizes are needed to assess whether interindividual differences in incongruency‐specific PD effects in YAs can identify different motivational levels or task strategies.

While MCI patients showed worse accuracy and RTs than OAs across congruent and incongruent conditions, PD responses in the Simon task were similar between groups. Despite small sample sizes requiring cautious interpretation (Figures S4 and S5 in the Supplement), as in the oddball task, some consistent patterns emerged: Individuals with faster RTs generally showed larger PDs, both on correct and incorrect incongruent trials (Figures S8 and S9 in Supplement), as well as on the differences in PD correct incongruent–correct congruent (Figure S10 in the Supplement), reflecting a more specific measure of increased PD due to increased attentional and cognitive control. This was rarely the case for OAs, suggesting less heterogeneity in mobilizing additional cognitive resources. MCI patients were the only group with sufficient variance in cognitive dementia screenings for examining interindividual differences. Higher accuracy and lower RTs in the Simon task were linked to better memory scores, underlining the functional relevance of examining executive function in MCI. This dissociation may indicate that basic attentional capacities remain relatively intact in the early stages of MCI, as suggested by preserved PD patterns, or that more targeted or sensitive assessments are necessary to detect subtle changes in attention in individuals with MCI. Laboratory‐based attentional tasks coupled with pupillometry may be promising in capturing aspects of executive attention that brief clinical screenings cannot assess.

Unexpectedly, we observed increased dilation and constriction in the early pupillary processes after stimulus presentation in OAs and MCI in the Simon task. It is presently unclear what these early pupil reactions reflect and why they differ across age groups. If earlier time windows in pupil response reflect earlier attentional and sensory processes, as appears to be the case for electroencephalography data,^76^ this might indicate heightened sensory processing in older groups. Future studies should explore these early pupil reactions in varying sensory processing demands.

Finally, changes in PD were not associated with LC integrity on either task, despite lower LC integrity in MCI compared to YAs and OAs. Although the LC is a key norepinephrine hub involved in attentional regulation^3^, ^5^ and LC firing has been linked to increased PD,^13^, ^14^, ^22^ PD responses to salient stimuli may be influenced by multiple neural pathways beyond the noradrenergic LC system.^77^ Additionally, compensatory mechanisms in MCI may add complexity to functional correlates, obscuring direct relationships with LC integrity. Larger studies combining LC fMRI with concurrent PD measures are needed to determine whether and under which conditions PD can serve as an indicator of LC function in aging and MCI/AD. Notably, we observed that higher LC integrity was associated with faster RTs during correct incongruent trials in MCI patients, consistent with previous research linking LC integrity to cognitive task performance^42^, ^43^, ^44^, ^78^ and highlighting the relevance of LC decline in neurodegeneration.

Overall, our study demonstrates that PD measures of the Simon and oddball tasks can successfully index cognitive function changes related to executive and attentional processing in healthy aging and MCI. Consistent with our hypothesis, OAs and MCI patients showed a similar pattern of pupillometric results, with oddball stimuli and incongruent trials requiring more attention/effort than standard stimuli in the oddball task and congruent trials in the Simon task, respectively. The preservation of similar PD response patterns across conditions, coupled with similar correlation patterns across YAs, OAs, and MCI patients, suggests that basic neural mechanisms governing attentional resource allocation remain intact despite behavioral performance differences. This dissociation reveals the clinical value of pupillometry: It identifies preserved cognitive engagement mechanisms that cognitive testing and behavioral measures alone cannot detect. The correlation between PD and task performance within groups further suggests that MCI patients maintaining larger PD responses to task‐relevant stimuli may represent a subgroup with better‐preserved cognitive capacity, indicating a potential window for interventions that rely on attention and effort mobilization. Due to the minimal demands on participants and the non‐invasive nature of pupillometry, these patterns can be effectively exploited through PD measures in the early stages of neurodegeneration. This offers a practical tool for characterizing cognitive function, identifying candidates for targeted interventions, and tracking disease progression or treatment response through repeated assessments. Moreover, adding PD measurements to cognitive assessments can provide insights into the required attention or effort required for producing particular behavioral results.

## CONFLICT OF INTEREST STATEMENT

Authors AZ, CL, EK, GDF, SL, MD, MFC, NW, RJD, RH, ED, and DH have no conflicts of interest to declare. Author disclosures are available in the supporting information.

## DECLARATION OF INTEREST

The Max Planck Institute for Human Cognitive and Brain Sciences and Wellcome Centre for Human Neuroimaging have institutional research agreements with Siemens Healthcare. NW holds a patent on acquisition of MRI data during spoiler gradients (US 10,401,453 B2). NW was a speaker at an event organized by Siemens Healthcare and was reimbursed for travel expenses. MFC: Patent filing 2205139.5: “Head Immobilisation in MRI Head Coils” held jointly with David Carmichael, Simon Richardson, and Frederic Dick; patent filing 15/712,942: “Motion detection using navigators at no scan time cost” held jointly with Nikolaus Weiskopf, Daniel Papp, UCL Business Ltd, and Siemens Healthineers. MFC was supported for attending the ESMRMB 2025. ED reports serving as an advisor and lecturer for Lilly, Roche, and Eisai.

## CONSENT STATEMENT

Written informed consent was obtained from all participants and their carers prior to participation in the experiment.

## Supporting information



Supporting Information

Supporting Information

Supporting Information

Supporting Information

Supporting Information

Supporting Information

Supporting Information

Supporting Information

Supporting Information

Supporting Information

Supporting Information

Supporting Information

Supporting Information

Supporting Information

Supporting Information

Supporting Information

Supporting Information

Supporting Information

## References

[1] Aston‐Jones G , Cohen JD . An integrative theory of locus coeruleus‐norepinephrine function: adaptive gain and optimal performance. Annual Review of Neuroscience. 2005;28(1):403‐450. doi:10.1146/annurev.neuro.28.061604.135709 16022602

[2] Kahneman D , Beatty J . Pupil diameter and load on memory. Science. 1966;154(3756):1583‐1585. doi:10.1126/science.154.3756.1583 5924930

[3] Berridge CW , Waterhouse BD . The locus coeruleus–noradrenergic system: modulation of behavioral state and state‐dependent cognitive processes. Brain Research Reviews. 2003;42(1):33‐84. doi:10.1016/s0165-0173(03)00143-7 12668290

[4] Kamp SM , Donchin E . ERP and pupil responses to deviance in an oddball paradigm. Psychophysiology. 2014;52(4):460‐471. doi:10.1111/psyp.12378 25369764

[5] Sara SJ . The locus coeruleus and noradrenergic modulation of cognition. Nature Reviews Neuroscience. 2009;10(3):211‐223. doi:10.1038/nrn2573 19190638

[6] Beatty J . Task‐evoked pupillary responses, processing load, and the structure of processing resources. Psychological Bulletin. 1982;91(2):276‐292. doi:10.1037/0033-2909.91.2.276 7071262

[7] Hämmerer D , Schwartenbeck P , Gallagher M , FitzGerald THB , Düzel E , Dolan RJ . Older adults fail to form stable task representations during model‐based reversal inference. Neurobiology of Aging. 2019;74:90‐100. doi:10.1016/j.neurobiolaging.2018.10.009 30439597 PMC6338680

[8] Murphy PR , O'Connell RG , O'Sullivan M , Robertson IH , Balsters JH . Pupil diameter covaries with BOLD activity in human locus coeruleus. Human Brain Mapping. 2014;35(8):4140‐4154. doi:10.1002/hbm.22466 24510607 PMC6869043

[9] Granholm E , Asarnow RF , Sarkin AJ , Dykes KL . Pupillary responses index cognitive resource limitations. Psychophysiology. 1996;33(4):457‐461. doi:10.1111/j.1469-8986.1996.tb01071.x 8753946

[10] Piquado T , Isaacowitz D , Wingfield A . Pupillometry as a measure of cognitive effort in younger and older adults. Psychophysiology. 2010;47(3):560‐569. doi:10.1111/j.1469-8986.2009.00947.x 20070575 PMC2867103

[11] Naber M , Murphy P . Pupillometric investigation into the speed‐accuracy trade‐off in a visuo‐motor aiming task. Psychophysiology. 2019;57(3):e13499. doi:10.1111/psyp.13499 31736089 PMC7027463

[12] Yokoi A , Weiler J . Pupil diameter tracked during motor adaptation in humans. Journal of Neurophysiology. 2022;128(5):1224‐1243. doi:10.1152/jn.00021.2022 36197019 PMC9722266

[13] Joshi S , Li Y , Kalwani Rishi M , Gold Joshua I . Relationships between Pupil Diameter and Neuronal Activity in the Locus Coeruleus, Colliculi, and Cingulate Cortex. Neuron. 2016;89(1):221‐234. doi:10.1016/j.neuron.2015.11.028 26711118 PMC4707070

[14] Reimer J , McGinley MJ , Liu Y , et al. Pupil fluctuations track rapid changes in adrenergic and cholinergic activity in cortex. Nature Communications. 2016;7(1):13289. doi:10.1038/ncomms13289 PMC5105162 27824036

[15] Varazzani C , San‐Galli A , Gilardeau S , Bouret S . Noradrenaline and dopamine neurons in the reward/effort trade‐off: a direct electrophysiological comparison in behaving monkeys. Journal of Neuroscience. 2015;35(20):7866‐7877. doi:10.1523/jneurosci.0454-15.2015 25995472 PMC6795183

[16] Wang CA , Boehnke SE , White BJ , Munoz DP . Microstimulation of the monkey superior colliculus induces pupil dilation without evoking saccades. Journal of Neuroscience. 2012;32(11):3629‐3636. doi:10.1523/jneurosci.5512-11.2012 22423086 PMC6703448

[17] Alnaes D , Sneve MH , Espeseth T , Endestad T , van de Pavert SHP , Laeng B . Pupil size signals mental effort deployed during multiple object tracking and predicts brain activity in the dorsal attention network and the locus coeruleus. Journal of Vision. 2014;14(4):1‐1. doi:10.1167/14.4.1 24692319

[18] Xuan B , Mackie MA , Spagna A , et al. The activation of interactive attentional networks. NeuroImage. 2016;129:308‐319. doi:10.1016/j.neuroimage.2016.01.017 26794640 PMC4803523

[19] Bornert P , Bouret S . Locus coeruleus neurons encode the subjective difficulty of triggering and executing actions. Rushworth MFS , ed. PLOS Biology. 2021;19(12):e3001487. doi:10.1371/journal.pbio.3001487 34874935 PMC8683033

[20] Ghosh S , Maunsell JHR . Locus coeruleus norepinephrine contributes to visual‐spatial attention by selectively enhancing perceptual sensitivity. Neuron. 2024;112(13):2231‐2240.e5. doi:10.1016/j.neuron.2024.04.001 38701788 PMC11223979

[21] Xiang L , Harel A , Gao H , Pickering AE , Sara SJ , Wiener SI . Behavioral correlates of activity of optogenetically identified locus coeruleus noradrenergic neurons in rats performing T‐maze tasks. Scientific Reports. 2019;9(1):1361. doi:10.1038/s41598-018-37227-w 30718532 PMC6362200

[22] de Gee JW , Colizoli O , Kloosterman NA , Knapen T , Nieuwenhuis S , Donner TH . Dynamic modulation of decision biases by brainstem arousal systems. Stephan KE , ed. eLife. 2017;6:e23232. doi:10.7554/eLife.23232 28383284 PMC5409827

[23] Lloyd B , de D , Mäki‐Marttunen V , Nieuwenhuis S . Pupil size reflects activation of subcortical ascending arousal system nuclei during rest. eLife. 2023;12:e84822. doi:10.7554/elife.84822 37367220 PMC10299825

[24] Grueschow M , Kleim B , Ruff CC . Role of the locus coeruleus arousal system in cognitive control. Journal of Neuroendocrinology. 2020;32(12):e12890. doi:10.1111/jne.12890 32820571

[25] Reuter‐Lorenz PA , Lustig C . Brain aging: reorganizing discoveries about the aging mind. Current Opinion in Neurobiology. 2005;15(2):245‐251. doi:10.1016/j.conb.2005.03.016 15831410

[26] de Gee JW , Tsetsos K , Schwabe L , et al. Pupil‐linked phasic arousal predicts a reduction of choice bias across species and decision domains. Gold JI , Grüschow M , Ebitz RB , eds. eLife. 2020;9:e54014. doi:10.7554/eLife.54014 32543372 PMC7297536

[27] Hämmerer D , Hopkins A , Betts MJ , Maaß A , Dolan RJ , Düzel E . Emotional arousal and recognition memory are differentially reflected in pupil diameter responses during emotional memory for negative events in younger and older adults. Neurobiology of Aging. 2017;58:129‐139. doi:10.1016/j.neurobiolaging.2017.06.021 28734217

[28] Granholm EL , Panizzon MS , Elman JA , et al. Pupillary responses as a biomarker of early risk for Alzheimer's disease. Journal of Alzheimer's Disease. 2017;56(4):1419‐1428. doi:10.3233/jad-161078 PMC5808562 28157098

[29] Podlasek A , Jastrzębski K . Entering the new era of cognitive scoring? eye‐tracking assessment in neurodegenerative disorders. Aktualności Neurologiczne. 2019;19(1):3‐7. doi:10.15557/an.2019.0001

[30] Porter G , Leonards U , Wilcock G , Haworth J , Troscianko T , Tales A . New insights into feature and conjunction search: II. evidence from Alzheimer's disease. Cortex. 2010;46(5):637‐649. doi:10.1016/j.cortex.2009.04.014 19595301

[31] Dragan MC , Leonard TK , Lozano AM , et al. Pupillary responses and memory‐guided visual search reveal age‐related and Alzheimer's‐related memory decline. Behavioural Brain Research. 2017;322:351‐361. doi:10.1016/j.bbr.2016.09.014 27616343

[32] Fletcher PD , Nicholas JM , Downey LE , et al. A physiological signature of sound meaning in dementia. Cortex. 2016;77:13‐23. doi:10.1016/j.cortex.2016.01.007 26889604 PMC4819950

[33] Opwonya J , Kim K , Lee KH , Kim JI , Kim JU . Task‐evoked pupillary responses as potential biomarkers of mild cognitive impairment. Alzheimer's & Dementia (Amsterdam, Netherlands). 2024;16(4): e70019. doi:10.1002/dad2.70019 PMC11465026 39391021

[34] Corbo I , Casagrande M . Higher‐Level Executive functions in healthy elderly and mild cognitive impairment: a systematic review. Journal of Clinical Medicine. 2022;11(5):1204. doi:10.3390/jcm11051204 35268294 PMC8911402

[35] Guarino A , Favieri F , Boncompagni I , Agostini F , Cantone M , Casagrande M . Executive functions in Alzheimer disease: a systematic review. Frontiers in Aging Neuroscience. 2019;10(437):437. doi:10.3389/fnagi.2018.00437 30697157 PMC6341024

[36] Kirova AM , Bays RB , Lagalwar S . Working memory and executive function decline across normal aging, mild cognitive impairment, and Alzheimer's disease. BioMed Research International. 2015;2015:1‐9. doi:10.1155/2015/748212 PMC4624908 26550575

[37] Clark LR , Schiehser DM , Weissberger GH , Salmon DP , Delis DC , Bondi MW . Specific measures of executive function predict cognitive decline in older adults. Journal of the International Neuropsychological Society. 2012;18(1):118‐127. doi:10.1017/S1355617711001524 22115028 PMC3314335

[38] Reinvang I , Grambaite R , Espeseth T . Executive dysfunction in mci: subtype or early symptom. International Journal of Alzheimer's Disease. 2012;2012:1‐8. doi:10.1155/2012/936272 PMC3369514 22693679

[39] Van Dam NT , Sano M , Mitsis EM , et al. Functional neural correlates of attentional deficits in amnestic mild cognitive impairment. Ginsberg SD , ed. PLoS ONE. 2013;8(1):e54035. doi:10.1371/journal.pone.0054035 23326568 PMC3543395

[40] Jiménez EC , Sierra‐Marcos A , Romeo A , et al. Altered vergence eye movements and pupil response of patients with alzheimer's disease and mild cognitive impairment during an oddball task. Journal of Alzheimer's Disease. Published online May 16, 2021:1‐13. doi:10.3233/jad-201301 34024820

[41] Luo C , Proctor RW . How different direct association routes influence the indirect route in the same Simon‐like task. Psychological Research. 2018;83(8):1733‐1748. doi:10.1007/s00426-018-1024-5 29761377

[42] Dahl MJ , Mather M , Düzel S , et al. Rostral locus coeruleus integrity is associated with better memory performance in older adults. Nat Hum Behav. 2019;3(11):1203‐1214. doi:10.1038/s41562-019-0715-2 31501542 PMC7203800

[43] Hämmerer D , Callaghan MF , Hopkins A , et al. Locus coeruleus integrity in old age is selectively related to memories linked with salient negative events. Proc Natl Acad Sci U S A. 2018;115(9):2228‐2233. doi:10.1073/pnas.1712268115 29440429 PMC5834676

[44] Liu KY , Kievit RA , Tsvetanov KA , et al. Noradrenergic‐dependent functions are associated with age‐related locus coeruleus signal intensity differences. Nature Communications. 2020;11(1):1712. doi:10.1038/s41467-020-15410-w PMC7136271 32249849

[45] de Gee JW , Knapen T , Donner TH . Decision‐related pupil dilation reflects upcoming choice and individual bias. Proceedings of the National Academy of Sciences. 2014;111(5):E618‐E625. doi:10.1073/pnas.1317557111 PMC3918830 24449874

[46] Cloutier S , Chertkow H , Kergoat MJ , Gauthier S , Belleville S . Patterns of cognitive decline prior to dementia in persons with mild cognitive impairment. Bondi M , ed. Journal of Alzheimer's Disease. 2015;47(4):901‐913. doi:10.3233/jad-142910 PMC4923749 26401770

[47] ElShafei HA , Masson R , Fakche C , et al. Age‐related differences in bottom‐up and top‐down attention: insights from EEG and MEG. European Journal of Neuroscience. 2022;55(5):1215‐1231. doi:10.1111/ejn.15617 35112420 PMC9303169

[48] Veríssimo J , Verhaeghen P , Goldman N , Weinstein M , Ullman MT . Evidence that ageing yields improvements as well as declines across attention and executive functions. Nature Human Behaviour. 2021;6:97‐110. doi:10.1038/s41562-021-01169-7 34413509

[49] Folstein MF , Folstein SE , McHugh PR . “Mini‐mental state”. A practical method for grading the cognitive state of patients for the clinician. Journal of Psychiatric Research. 1975;12(3):189‐198. doi:10.1016/0022-3956(75)90026-6 1202204

[50] van Steenbergen H , Band GPH . Pupil dilation in the Simon task as a marker of conflict processing. Frontiers in Human Neuroscience. 2013;7:215. doi:10.3389/fnhum.2013.00215 23754997 PMC3665936

[51] Nelson . The National Adult Reading Test (NART): Test Manual. NFER‐Nelson; 1982.

[52] Mathuranath PS , Nestor PJ , Berrios GE , Rakowicz W , Hodges JR . A brief cognitive test battery to differentiate Alzheimer's disease and frontotemporal dementia. Neurology. 2000;55(11):1613‐1620. doi:10.1212/01.wnl.0000434309.85312.19 11113213

[53] Zigmond AS , Snaith RP . The Hospital Anxiety and Depression Scale. Acta Psychiatrica Scandinavica. 1983;67(6):361‐370. doi:10.1111/j.1600-0447.1983.tb09716.x 6880820

[54] Smyth C . The Pittsburgh Sleep Quality Index (PSQI). Journal of Gerontological Nursing. 1999;25(12):10‐10. doi:10.3928/0098-9134-19991201-10 10711108

[55] Bucks RS , Ashworth DL , Wilcock GK , Siegfried K . Assessment of activities of daily living in dementia: development of the Bristol activities of daily living scale. Age and Ageing. 1996;25(2):113‐120. doi:10.1093/ageing/25.2.113 8670538

[56] Alexopoulos GS , Abrams RC , Young RC , Shamoian CA . Cornell scale for depression in dementia. Biological Psychiatry. 1988;23(3):271‐284. doi:10.1016/0006-3223(88)90038-8 3337862

[57] Cummings JL , Mega M , Gray K , Rosenberg‐Thompson S , Carusi DA , Gornbein J . The neuropsychiatric inventory: comprehensive assessment of psychopathology in dementia. Neurology. 1994;44(12):2308‐2308. doi:10.1212/wnl.44.12.2308 7991117

[58] Mioshi E , Dawson K , Mitchell J , Arnold R , Hodges JR . The Addenbrooke's Cognitive Examination Revised (ACE‐R): a brief cognitive test battery for dementia screening. International Journal of Geriatric Psychiatry. 2006;21(11):1078‐1085. doi:10.1002/gps.1610 16977673

[59] Bouret S , Ravel S , Richmond BJ . Complementary neural correlates of motivation in dopaminergic and noradrenergic neurons of monkeys. Frontiers in Behavioral Neuroscience. 2012;6:40. doi:10.3389/fnbeh.2012.00040 22822392 PMC3398259

[60] Nassar MR , Rumsey KM , Wilson RC , Parikh K , Heasly B , Gold JI . Rational regulation of learning dynamics by pupil‐linked arousal systems. Nature Neuroscience. 2012;15(7):1040‐1046. doi:10.1038/nn.3130 22660479 PMC3386464

[61] MacDonald SWS , Li SC , Bäckman L . Neural underpinnings of within‐person variability in cognitive functioning. Psychology and Aging. 2009;24(4):792‐808. doi:10.1037/a0017798 20025396

[62] Betts MJ , Cardenas‐Blanco A , Kanowski M , Jessen F , Düzel E . In vivo MRI assessment of the human locus coeruleus along its rostrocaudal extent in young and older adults. NeuroImage. 2017;163:150‐159. doi:10.1016/j.neuroimage.2017.09.042 28943414

[63] Keren NI , Taheri S , Vazey EM , et al. Histologic validation of locus coeruleus MRI contrast in post‐mortem tissue. NeuroImage. 2015;113:235‐245. doi:10.1016/j.neuroimage.2015.03.020 25791783 PMC4649944

[64] Betts MJ , Kirilina E , Otaduy MCG , et al. Locus coeruleus imaging as a biomarker for noradrenergic dysfunction in neurodegenerative diseases. Brain. 2019;142(9):2558‐2571. doi:10.1093/brain/awz193 31327002 PMC6736046

[65] Yushkevich PA , Piven J , Hazlett HC , et al. User‐guided 3D active contour segmentation of anatomical structures: significantly improved efficiency and reliability. Neuroimage. 31(3):1116‐1128. doi:10.1016/j.neuroimage.2006.01.015 16545965

[66] Liu KY , Marijatta Freya , Hämmerer D , Acosta‐Cabronero J , Düzel Emrah , Howard R . Magnetic resonance imaging of the human locus coeruleus: a systematic review. Neurosci Biobehav Rev. 2017;83:325‐355. doi:10.1016/j.neubiorev.2017.10.023 29107830

[67] Clewett DV , Lee TH , Greening S , Ponzio A , Margalit E , Mather M . Neuromelanin marks the spot: identifying a locus coeruleus biomarker of cognitive reserve in healthy aging. Neurobiology of aging. 2016;37:117‐126. doi:10.1016/j.neurobiolaging.2015.09.019 26521135 PMC5134892

[68] Keren NI , Lozar CT , Harris KC , Morgan PS , Eckert MA . In vivo mapping of the human locus coeruleus. NeuroImage. 2009;47(4):1261‐1267. doi:10.1016/j.neuroimage.2009.06.012 19524044 PMC3671394

[69] R Core Team . R: A language and environment for statistical computing. R Foundation for Statistical Computing, Vienna, Austria. 2024. http://www.R‐project.org/

[70] Liu KY , Acosta‐Cabronero J , Cardenas‐Blanco A , et al. In vivo visualization of age‐related differences in the locus coeruleus. Neurobiology of Aging. 2019;74:101‐111. doi:10.1016/j.neurobiolaging.2018.10.014 30447418 PMC6338679

[71] Yeung N , Summerfield C . Shared Mechanisms for Confidence Judgements and Error Detection in Human Decision Making. Springer eBooks. Published online January 1, 2014:147‐167. doi:10.1007/978-3-642-45190-4_7

[72] Ghayedi Z , Banihashemian K , Shirdel S , et al. A Review of the comparison of working memory performance, cognitive function, and behavioral, and psychological symptoms across normal aging, mild cognitive impairment, and Alzheimer's disease. Neurology Letters. 2024;3(Special Issue):26‐38. doi:10.61186/nl.3.2.26

[73] Gilzenrat MS , Nieuwenhuis S , Jepma M , Cohen JD . Pupil diameter tracks changes in control state predicted by the adaptive gain theory of locus coeruleus function. Cognitive, Affective, & Behavioral Neuroscience. 2010;10(2):252‐269. doi:10.3758/cabn.10.2.252 PMC3403821 20498349

[74] Simon JR , Small AM . Processing auditory information: interference from an irrelevant cue. Journal of Applied Psychology. 1969;53(5):433‐435. doi:10.1037/h0028034 5366316

[75] Salthouse TA . The processing‐speed theory of adult age differences in cognition. Psychological Review. 1996;103(3):403‐428. doi:10.1037/0033-295x.103.3.403 8759042

[76] Demeter E , Glassberg B , Gamble ML , Woldorff MG . Reward magnitude enhances early attentional processing of auditory stimuli. Cognitive, Affective, & Behavioral Neuroscience. Published online November 22, 2021. doi:10.3758/s13415-021-00962-1 PMC8986595 34811706

[77] Mathôt S . Pupillometry: psychology, physiology, and function. Journal of Cognition. 2018;1(1):16. doi:10.5334/joc.18 31517190 PMC6634360

[78] Smegal LF , Baillet M , Schneider C , et al. Lower locus coeruleus integrity is associated with diminished practice effects in clinically unimpaired older individuals. Neurobiology of Aging. 2025;152:13‐24. doi:10.1016/j.neurobiolaging.2025.03.015 40315539 PMC12581611

