# Supplementary material for: Pupil dilation as a marker of attention/effort in aging and mild cognitive impairment
Source: Alzheimers Dement. 2026 Mar 13;22(3):e71180. doi: 10.1002/alz.71180 (PMC13093636; doi:10.1002/alz.71180)
Supplement: Supplementary file 9 — Supporting Information [file ALZ-22-e71180-s017.pdf]

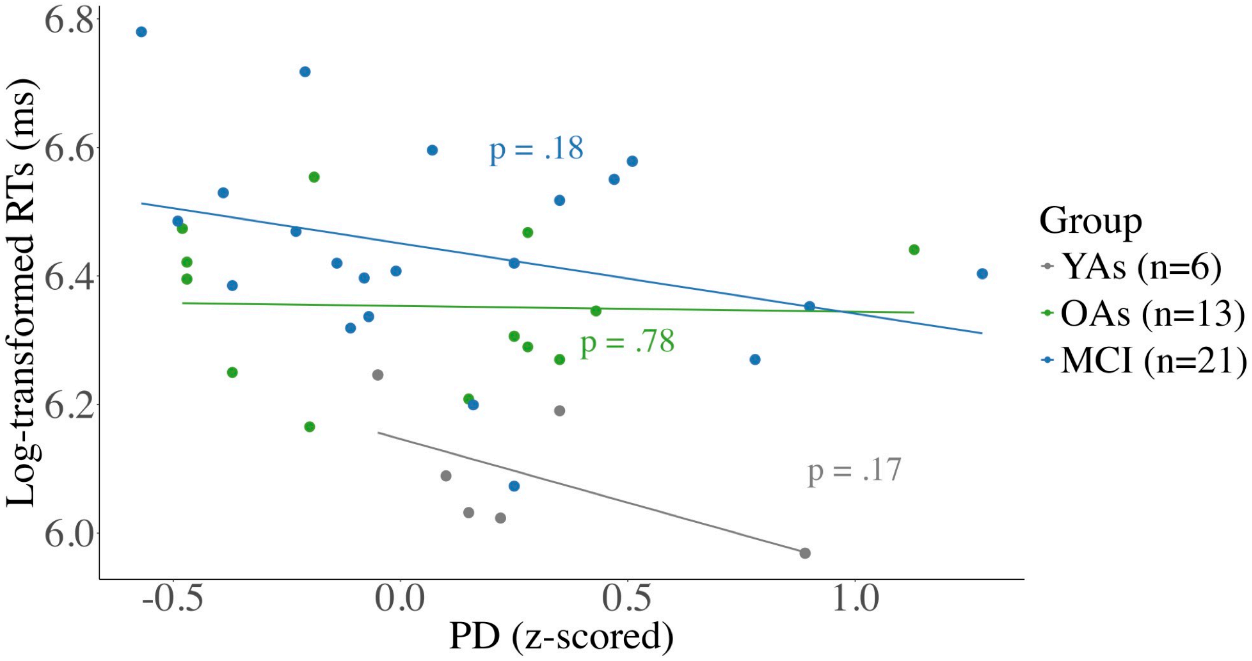

Figure S7. Spearman correlation between pupil dilation (PD) for the **standard stimuli** and hit reaction times (RTs) across groups in the **visual oddball task.** YAs – younger adults, OAs – older adults, MCI – patients with mild cognitive impairment.
