# Supplementary material for: Pupil dilation as a marker of attention/effort in aging and mild cognitive impairment
Source: Alzheimers Dement. 2026 Mar 13;22(3):e71180. doi: 10.1002/alz.71180 (PMC13093636; doi:10.1002/alz.71180)
Supplement: Supplementary file 10 — Supporting Information [file ALZ-22-e71180-s008.pdf]

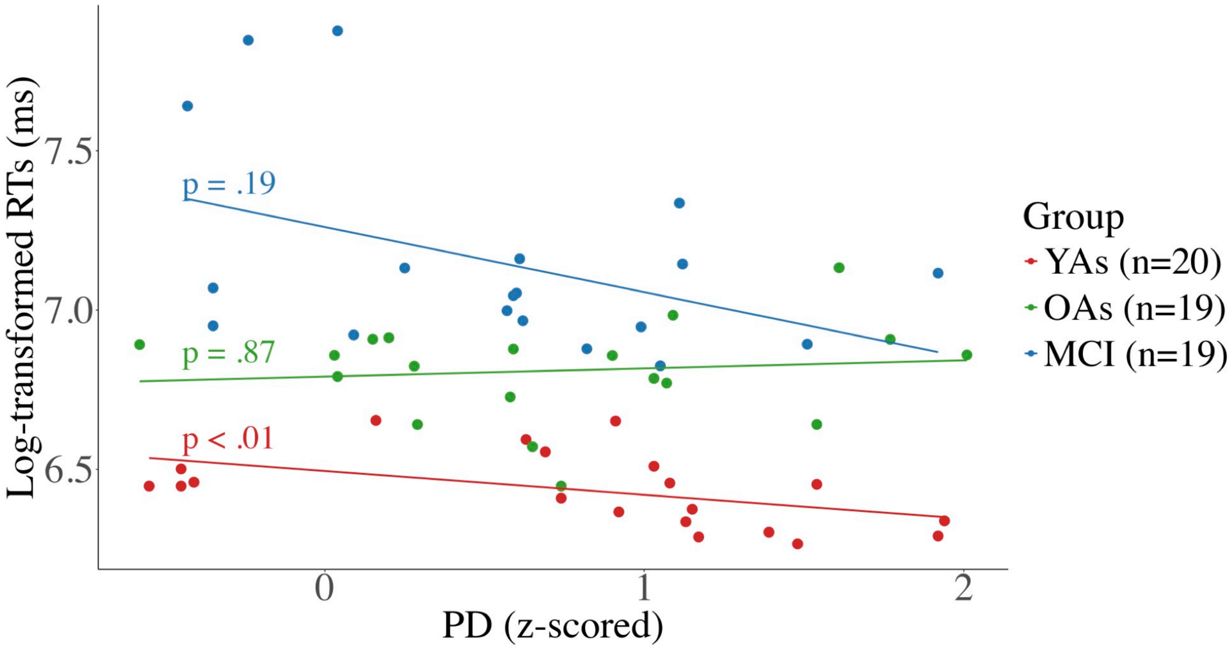

Figure S8. Spearman correlation between pupil dilation (PD) and reaction times (RTs) in correct incongruent trials across groups in the Simon task. YAs – younger adults, OAs – older adults, MCI – patients with mild cognitive impairment.
