# Supplementary material for: Pupil dilation as a marker of attention/effort in aging and mild cognitive impairment
Source: Alzheimers Dement. 2026 Mar 13;22(3):e71180. doi: 10.1002/alz.71180 (PMC13093636; doi:10.1002/alz.71180)
Supplement: Supplementary file 14 — Supporting Information [file ALZ-22-e71180-s014.pdf]

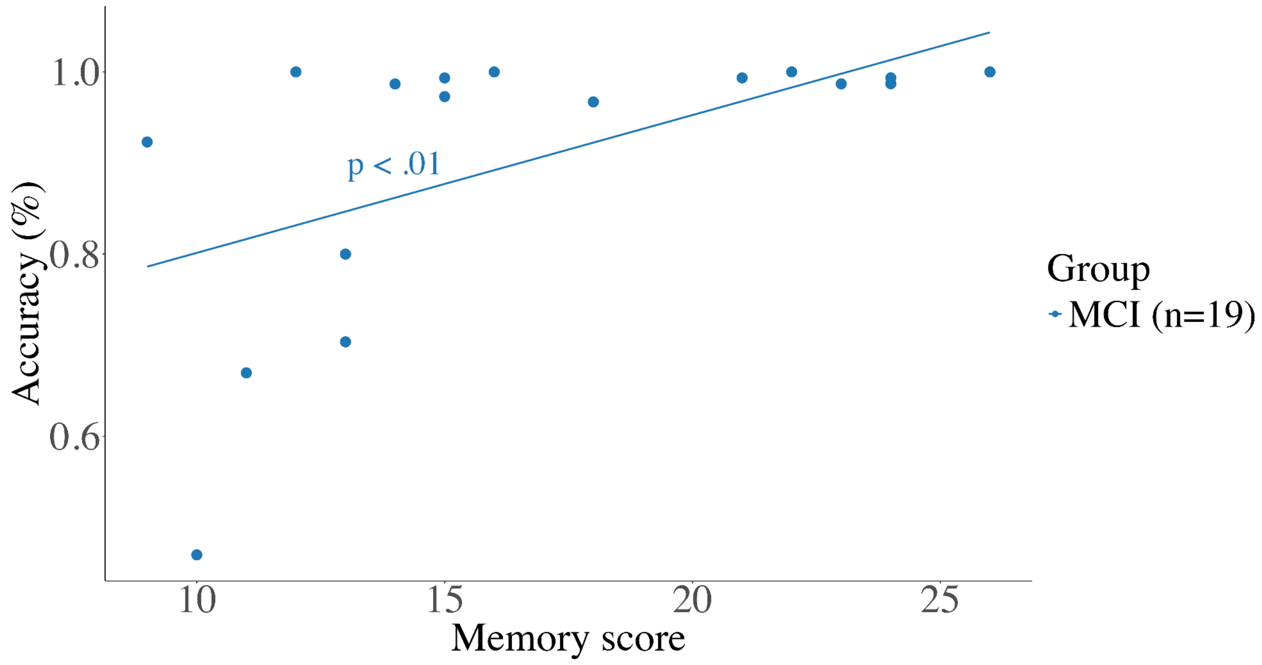

Figure S12. Spearman correlation between memory score in the revised version of Addenbrooke's Cognitive Examination and accuracy in correct congruent trials in the Simon task. MCI – patients with mild cognitive impairment.
