# Supplementary material for: Pupil dilation as a marker of attention/effort in aging and mild cognitive impairment
Source: Alzheimers Dement. 2026 Mar 13;22(3):e71180. doi: 10.1002/alz.71180 (PMC13093636; doi:10.1002/alz.71180)
Supplement: Supplementary file 17 — Supporting Information [file ALZ-22-e71180-s016.pdf]

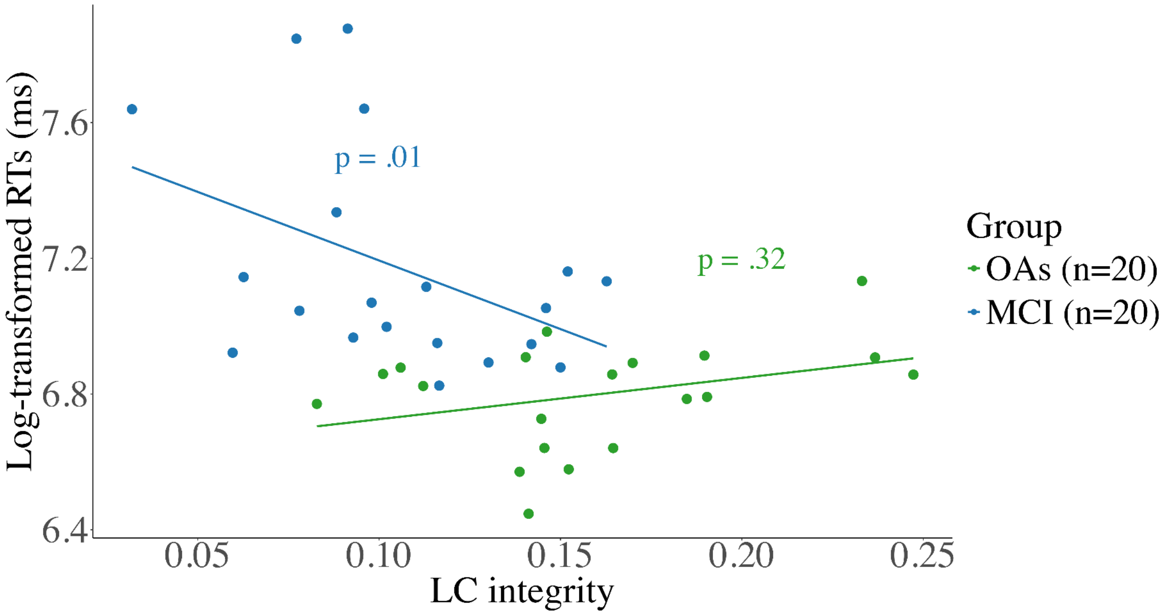

Figure S15. Relationship between locus coeruleus (LC) integrity and reaction times (RTs) in correct incongruent trials across groups in the Simon task. OAs – older adults, MCI – patients with mild cognitive impairment.
